# Shifting the substrate scope of dimeric pyranose oxidase from monosaccharide to glycoside preference through oligomeric state modification

**DOI:** 10.1111/febs.70004

**Published:** 2025-02-06

**Authors:** Anja Kostelac, Enikő Hermann, Clemens Peterbauer, Chris Oostenbrink, Dietmar Haltrich

**Affiliations:** ^1^ Department of Food Science and Technology BOKU University Vienna Austria; ^2^ Doctoral Programme BioToP – Biomolecular Technology of Proteins BOKU University Vienna Austria; ^3^ Department of Material Science and Life Sciences BOKU University Vienna Austria; ^4^ Christian Doppler Laboratory for Molecular Informatics in the Biosciences BOKU University Vienna Austria

**Keywords:** *C*‐glycoside oxidase, oligomerization, pyranose oxidase, rational engineering, substrate scope

## Abstract

Pyranose oxidase (POx) and *C*‐glycoside oxidase (CGOx) are FAD‐dependent oxidoreductases belonging to the glucose‐methanol‐choline oxidoreductase superfamily and share the same sequence space. Despite a shared structural fold, these two members possess homologous domains that enable (arm and head domain) or disable (insertion‐1 domain and barrel‐shaped bottom) oligomerization. POxs with a higher oligomerization state (dimeric or tetrameric) exclusively catalyze the oxidation of monosaccharides (d‐glucose, d‐xylose). In contrast, the monomeric state of POxs/CGOxs is observed to prefer glycosides (homoorientin, phlorizin) and has low activity with free monosaccharides. We aimed to engineer dimeric POx from *Kitasatospora aureofaciens* (*Ka*POx) to form a functional monomer, and monomeric POx/CGOx from *Streptomyces canus* (*Sc*POx) to a dimeric structure. Deletion of the head and arm domains of the *Ka*POx subunit resulted in enzyme variants with a less hydrophobic surface, thus affecting its oligomerization. These monomeric *Ka*POx variants *Ka*POx_xal and *Ka*POx_xalh resembled monomeric wild‐type POxs/CGOxs and preferred glycosides as substrates over monosaccharides with catalytic efficiencies for phlorizin being 24 × 10^6^ higher compared to those for d‐xylose. The wild‐type dimeric *Ka*POx showed no activity towards glycosides. We hypothesize that *Ka*POx_xalh is unable to react with monosaccharides because the introduced mutations alter the positions of monosaccharide‐binding residues. The inability of *Ka*POx to react with glycosides is likely caused by steric hindrance and the inaccessibility of the active site to bulky glycosides due to dimerization. The attempt to engineer *Sc*POx into a dimeric structure failed at the stage of soluble expression, likely due to exposed hydrophobic patches and aggregation.

## Graphical abstract

Pyranose oxidase (POx) and *C*‐glycoside oxidase (CGOx) are FAD‐dependent oxidoreductases that share the same sequence space. POxs with a higher oligomerization state exclusively catalyze the oxidation of monosaccharides, whereas monomeric POxs/CGOxs show preference towards glycosides. We engineered the dimeric POx from *Kitasatospora aureofaciens* (*Ka*POx) to form a monomer. Deletion of the oligomerization domains of *Ka*POx resulted in monomeric variants, which resemble bacterial wild‐type POxs/CGOxs and prefer glycosides as substrates.

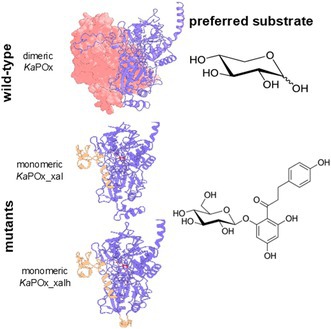

**Abbreviations**
CGOx: *C*‐glycoside oxidase
DCIP: 2,6‐dichlorophenolindophenol
DMSO: dimethyl sulfoxide
FAD: flavin adenine dinucleotide
GlycDH: *C*‐glycoside oxidase from *Rhizobium* sp. GIN611
GPC/SEC: gel permeation chromatography/size‐exclusion chromatography system
IMAC: immobilized metal affinity chromatography
IPTG: isopropyl β‐d‐1‐thiogalactopyranoside
*Ka*POx: pyranose oxidase from *Kitasatospora aureofaciens*
*Ka*POx_xal: *Ka*POx mutant without the oligomerization loop and the arm domain
*Ka*POx_xalh: *Ka*POx_xal mutant without the head domain
*Mt*CarA: FAD‐dependent *C*‐glycoside 3‐oxidase from *Microbacterium trichothecenolyticum*
*Pc*POx: pyranose oxidase from *Phanerochaete chrysosporium*
POx: pyranose oxidase
*Ps*POx: pyranose oxidase from *Pseudarthrobacter siccitolerans*
r.p.m.: revolutions per minute
*Sc*POx: pyranose oxidase from *Streptomyces canus*
SDS/PAGE: sodium dodecyl sulphate/polyacrylamide gel electrophoresis
SEC: size‐exclusion chromatography
SEC‐LS: size‐exclusion chromatography‐light scattering
*Tm*POx: pyranose oxidase from *Trametes multicolor*

## Introduction

How proteins interact at a molecular level is an intricate question. During evolution, a large diversity of proteins developed based on different folds, unique multimeric interaction, and modes of allosteric regulation. Many proteins assemble into complexes—either homomers, complexes made out of identical subunits with each chain having the same interactions within the complex, or heteromers, composed of two or more distinct polypeptide chains—to attain various and complex functions [1, 2, 3, 4]. Oligomerization allows the formation of large structures without increasing genome size and providing genome stability, and, at the same time, increases the potential functions of a protein [5, 6, 7]. Interestingly enough, most monomers contain large hydrophobic patches on their surface, which provide a foundation for new multimeric interactions [7, 8], and most interactions between subunits originate from non‐covalent hydrophobic effect, and only to a smaller extent from hydrogen bonds [4].

Pyranose oxidase (POx; pyranose 2‐oxidase, glucose 2‐oxidase; EC1.1.3.10, pyranose:oxygen 2‐oxidoreductase) and *C*‐glycoside oxidase (CGOx; *C*‐glycoside 3‐oxidase; EC1.1.3.50, carminate:oxygen 3′‐oxidoreductase (H_2_O_2_‐forming)) are members of the glucose‐methanol‐choline (GMC) oxidoreductase superfamily and share the same sequence space [9, 10, 11]. These flavin adenine dinucleotide (FAD)‐containing enzymes are thought to act as mediators between plants and microbes, catalyzing reactions involving plant‐derived molecules such as monosaccharides (d‐glucose, d‐galactose, d‐xylose) and glycosides (carminic acid, isoorientin, isovitexin, mangiferin, phlorizin) [10, 12, 13, 14, 15]. The role of monosaccharide‐oxidizing POx has mainly been seen as an accessory enzyme in lignocellulose deconstruction by providing hydrogen peroxide to peroxidases [12, 16], whereas glycoside‐oxidizing CGOx initiates the cleavage of *C*‐ and *O*‐glycosides [14, 15, 17]. Although both POxs and CGOxs share the same overall catalytic reaction, i.e., the oxidation of a sugar moiety at its C2 or C3 position, a number of different POxs or CGOxs have been reported, each with its distinctive set of biochemical properties. The first POxs discovered, such as the fungal POx from *Trametes multicolor* (*Tm*POx), are homotetramers (~ 270 kDa, more specifically dimers of dimers with a monomer mass of ~ 65 kDa) with a strong preference towards the oxidation of monosaccharides [16, 18, 19, 20]. A closely related bacterial POx from *Kitasatospora aureofaciens* (*Ka*POx) shares a similar substrate preference, however is reported to have a dimeric structure (120 kDa, subunit size of ~ 61 kDa) [12]. In contrast, all other bacterial POxs/CGOxs reported since 2021, such as POx/CGOx from *Streptomyces canus* (*Sc*POx), show distinct preference for glycosides as their substrates and have monomeric structures (55–60 kDa) [10, 14, 15].

In a recent study, we investigated the actinobacterial POx/CGOx sequence space, which is formed by four distinct clades. Clade I contains *Ka*POx, no extant member of clade II has been studied to date, and clades III and IV contain several studied, monomeric enzymes oxidizing preferentially glycosides [21]. Based on the inference of ancestors at various nodes of clade I and comparison with the common ancestor of the actinobacterial POx/CGOx group, we hypothesized that clade I POxs modified their substrate range by altering the oligomeric state during evolution from monomers to dimers [21]. We postulated that during evolution, POxs/CGOxs underwent changes in such a manner that oligomeric enzymes restrict the entrance to their active sites to small substrates (such as monosaccharides). In contrast, enzymes existing as monomers preferentially oxidize bigger substrates (such as glycosides).

In this study, we aimed at engineering a monomeric bacterial POx/CGOx into a dimer, and a dimeric bacterial POx into a monomer. We chose *Ka*POx as the template for dimers and *Sc*POx as the template for monomers. Through altering the oligomeric state, our objective was to validate the hypothesis that oligomerization affects the substrate scope of POx/CGOx.

## Results

### Design of *Ka*POx and *Sc*POx variants

**Fig. 1:**
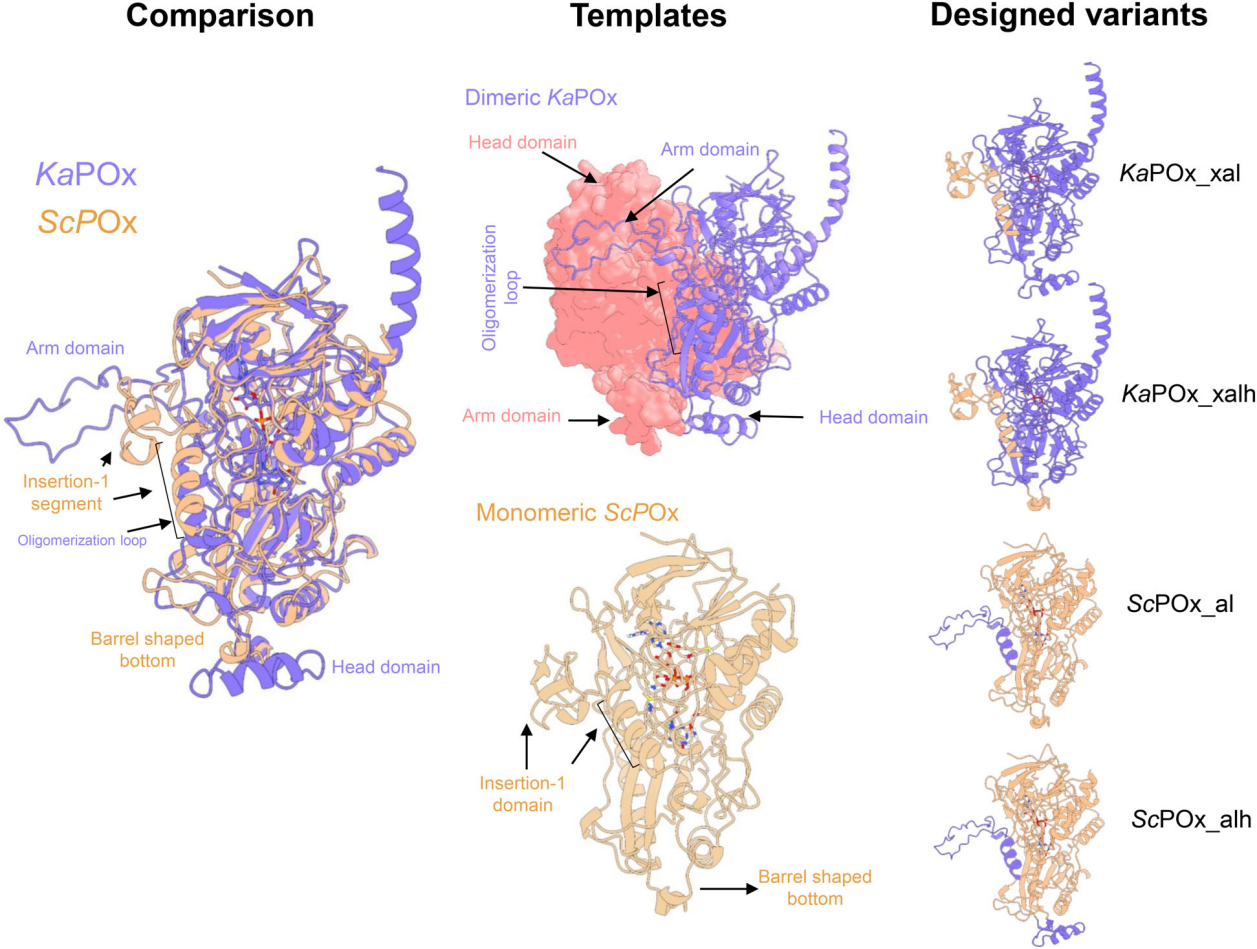
Schematic design of *Ka*POx and *Sc*POx variants. Structural models of dimeric *Ka*POx (purple/pink) and monomeric *Sc*POx (yellow), including their comparison with annotated structural motifs presumed to enable or disable homooligomerization (first and second column). In the dimeric representation of *Ka*POx, one subunit is displayed as cartoon (purple), while the other as surface model (pink). Furthermore, illustrations depicting the designed variants *Ka*POx_xal, *Ka*POx_xalh, *Sc*POx_al, and *Sc*POx_alh through schematic representations, accompanied by an explanation of the design process in the third column. FAD is represented as a ball‐and‐stick model within each variant.

A structural prediction of dimeric *Ka*POx portrays a similar fold as a dimer of *Tm*POx (PDB 1TT0), with well‐defined arm and head domains as well as an oligomerization loop (Fig. 1), which play a role in dimerization [12]. Monomeric POxs, based on the crystal structures of *Pseudarthrobacter siccitolerans* POx (*Ps*POx) and FAD‐dependent *C*‐glycoside 3‐oxidase from *Microbacterium trichothecenolyticum* (*Mt*CarA), show deletions in their primary structure that correspond to the oligomerization loop, the arm and the head domain [10, 14]. The *Ps*POx crystal structure (7QF8), however, displays the insertion‐1 segment, a flexible loop that corresponds to the position of the oligomerization loop and arm domain. A barrel‐shaped bottom of the enzymes has also been described [21].

A sequence alignment of *Ka*POx and *Sc*POx reveals that despite a sequence identity of only 33%, structural motifs characteristic for dimeric POxs – arm domain, oligomerization loop and head domain – and characteristic motifs of monomeric POxs – the insertion‐1 segment and the barrel‐shaped bottom – correspond to identical regions in the primary sequence (Fig. S1). When comparing the length of the arm domain and the oligomerization loop with that of the insertion‐1 loop, and that of the head domain and the barrel‐shaped bottom, it is evident that these regions are extended by 28 and 26 additional amino acids, respectively, in dimeric *Ka*POx. However, when comparing these regions by superimposition of structural models, they align well with different folds taking up comparable positions (Fig. 1). Upon inspection of the interactions between the two *Ka*POx monomers in the functional dimer, the arm domain of one monomer interacts with a certain segment of the head domain of the other subunit, and vice versa (Fig. 1, second column, top panel). The oligomerization loop was observed to be involved in the subunit interaction as well, mainly through interaction with the other oligomerization loop. These corresponding regions in *Sc*POx, the insertion‐1 segment and the barrel‐shaped bottom, are much smaller and hence cannot contribute to comparable interactions (Fig. 1, second column, bottom panel).

Based on these observations, suggesting the importance of these regions in bacterial POx, we designed four variants (Fig. 1, right column);

- a *Ka*POx variant with both a deleted arm domain and oligomerization loop, which were substituted by the insertion‐1 domain of *Sc*POx and the flexible loop just before the insertion‐1 domain of *Sc*POx (*Ka*POx_xal),
- a *Ka*POx variant that is identical to the first *Ka*POx variant with an additionally deleted head domain, substituted by the barrel‐shaped bottom of *Sc*POx (*Ka*POx_xalh),
- an *Sc*POx variant with both a deleted insertion‐1 domain and flexible loop, which were substituted by the arm domain and the oligomerization loop of *Ka*POx, respectively (*Sc*POx_al), and
- an *Sc*POx variant that is identical to the first *Sc*POx variant with an additionally deleted barrel‐shaped bottom, substituted by the head domain of *Ka*POx (*Sc*POx_alh) (Fig. 1).

Variants (i) and (ii) were designed to investigate whether the *Ka*POx dimer can be converted into a functional monomer, while the changes in (iii) and (iv) should convert *Sc*POx into a dimer. No additional alterations were made to the sequences, ensuring that both the substrate loop, a loop that allows entrance of the substrate into the active site, and the active‐site residues, residues that form interactions with the sugar substrate, remained unchanged. Primary sequences of these variants are shown in Table S1.

### Purification and biochemical properties of recombinant *Ka*POx variants

An initial screening for expression of the *kapox_xal* and *kapox_xalh* genes in the soluble cell extracts of *Escherichia coli* T7 Express showed bands corresponding to the size of the proteins of interest on SDS/PAGE (data not shown). In contrast, protein bands corresponding to the products of *scpox_al* and *scpox_alh* were not detected. Despite re‐cloning these latter genes into various constructs and transforming different *E. coli* expression hosts with the newly cloned constructs (with and without overexpressed chaperones), we did not observe corresponding protein bands in any of the soluble fractions of the *E. coli* cell extracts, but rather in the cell debris. Presumably, these two variants are formed in inclusion bodies. Therefore, we continued studying only *Ka*POx_xal and *Ka*POx_xalh.

Recombinantly produced *Ka*POx_xal showed more than 95% purity after purification with IMAC, whereas *Ka*POx_xalh was purified with an additional SEC step as well (Fig. S2). SEC‐LS measurements for the determination of the molecular masses of the purified proteins showed signals of 61 and 57 kDa (Fig. S3), indicating a monomeric state for both *Ka*POx_xal and *Ka*POx_xalh, respectively. The theoretical molecular masses of the *Ka*POx_xal and *Ka*POx_xalh monomers are 58.7 and 56.9 kDa. We also performed SEC‐LS measurements with different protein concentrations of *Ka*POx_xalh to investigate whether an equilibrium between monomers and dimers exists at higher protein concentrations. These results indicate that *Ka*POx_xalh is a stable monomer at concentrations ranging from 2 to 11 mg·mL^−1^ and that its oligomeric state is independent of concentration (Fig. S4). UV–Vis spectra of the purified protein preparations showed typical FAD peaks, confirming that the proteins contain FAD as their prosthetic group (Fig. S5). While the FAD loading was > 95% for *Ka*POx_xalh, *Ka*POx_xal exhibited FAD loading of only 21%. This incomplete loading was taken into account for the activity measurements and calculations. *Ka*POx_xal was expressed in much higher yields (~ 10 times higher) than *Ka*POx_xalh, yet the expression yield was still approximately three times lower than for wild‐type *Ka*POx (Table S2). Thermostability of *Ka*POx_xal and *Ka*POx_xalh as measured by the Thermo FAD assay was reduced by 11 °C compared to dimeric wild‐type *Ka*POx.

### Kinetic properties of *Ka*POx variants show a change of substrate preference

**Fig. 2:**
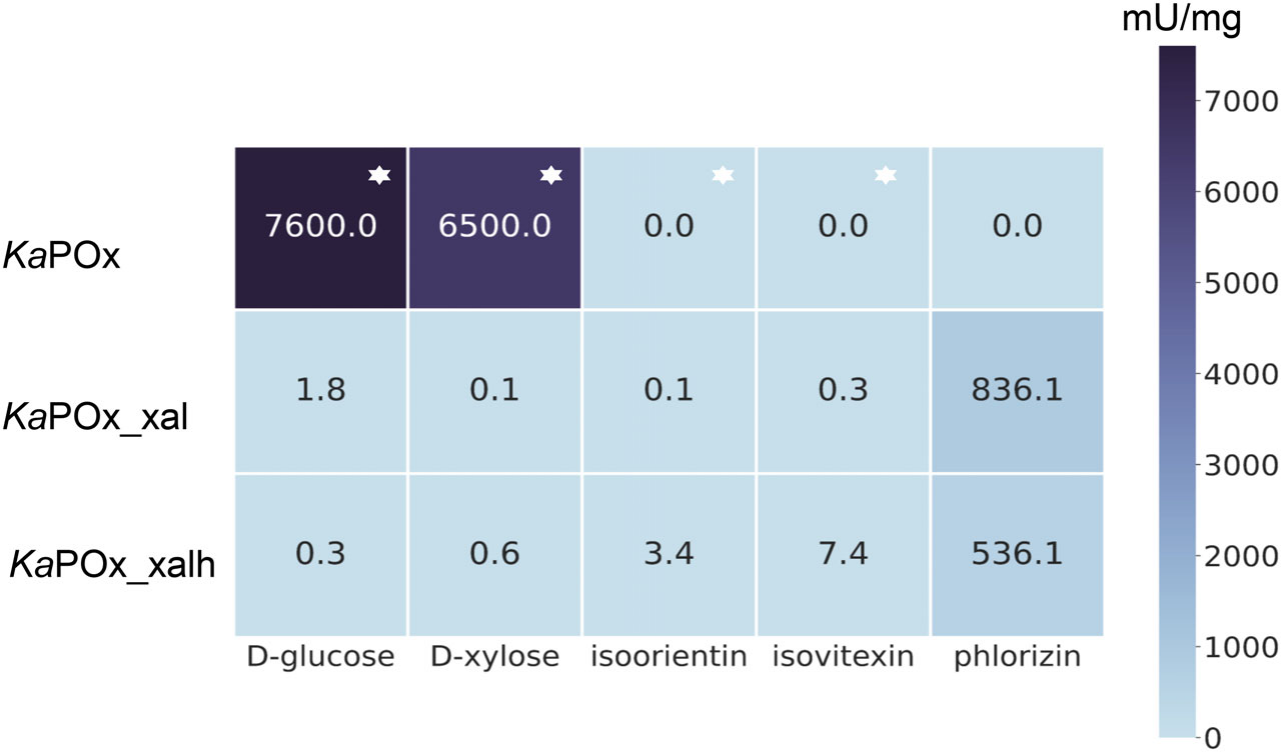
Substrate screening for dimeric *Ka*POx and monomeric *Ka*POx_xal and *Ka*POx_xalh. Heatmap showing specific activities (in mU·mg^−1^) during the initial screening for activity of dimeric *Ka*POx and monomeric *Ka*POx_xal and *Ka*POx_xalh. The concentrations of substrates were 200 mm for monosaccharides and 0.02 mm for glycosides. Measurements were performed in triplicate at 30 °C in 50 mm Tris/HCl, pH = 7.5. The values labeled with a white asterisk were obtained from the measured *v*_max_ values, whereas those with a gray asterisk from previous screening for activity. Both are taken from prior publications [12, 21].

Screening for activity was done with both the dehydrogenase (2,6‐dichlorophenolindophenol (DCIP) as electron acceptor in the oxidative half‐reaction) and oxidase (oxygen as electron acceptor) assays, however, only the oxidase assay with Amplex Red and oxygen/air gave positive results. The activity of wild‐type *Ka*POx was only tested using phlorizin as a glycoside substrate, as its activity with other substrates (d‐glucose, d‐xylose) has been studied, and it had not shown any activity with a range of different *C*‐glycosides [21]. This strict preference of wild‐type *Ka*POx was again confirmed since it did not oxidize phlorizin. The initial activity screening of *Ka*POx_xal and *Ka*POx_xalh showed activities with the monosaccharides d‐glucose and d‐xylose, as well as with the *C*‐glycosides isoorientin (homoorientin), isovitexin and the *O*‐glycoside phlorizin (Fig. 2). The structures of these different substrates are shown in Table S3. Potential substrates that were however not oxidized by the two variants included the *C*‐glycosides carminic acid, mangiferin and puerarin, the *O*‐glycosides fraxin, salicin, and rutin, and the *S*‐glycoside sinigrin. The monomeric variants *Ka*POx_xal and *Ka*POx_xalh exhibited activity for monosaccharides that is ~ 4000 and 25 000 times lower than that of the wild‐type. In contrast to wild‐type *Ka*POx, both enzymes exhibited considerable activity towards several glycosides, with phlorizin giving the highest values of 840 and 540 mU·mg^−1^, while the specific activities with isoorientin and isovitexin are considerably lower. We therefore selected those substrates yielding the highest activity for detailed kinetic characterization –d‐xylose as a representative substrate for monosaccharides and phlorizin for glycosides.

Apparent steady‐state constants show a clear difference between the different enzymes (Table 1). The corresponding Michaelis–Menten curves can be found in Fig. S6. Wild‐type *Ka*POx exhibits a preference for d‐xylose compared to its variants, with a catalytic efficiency of 210 m ^−1^·s^−1^, while *Ka*POx_xal and *Ka*POx_xalh display much lower efficiencies, 0.0031 m ^−1^·s^−1^ for *Ka*POx_xal. The constants for *Ka*POx_xalh could not be estimated properly as even 800 mm xylose in the assay did not approach substrate saturation. The decrease in catalytic efficiency in the variants primarily originates from a substantial decrease in *k*_cat_ (from 6.8 to 0.0013 s^−1^), and an unfavorable change in *K*_m_ (from 32 to 400 mm). In contrast, the catalytic efficiency of *Ka*POx_xal towards phlorizin is 5 × 10^4^ m ^−1^·s^−1^. This high catalytic efficiency is mainly the result the low *K*_m_ of approximately ~ 0.02 mm. When comparing the selectivity ratio (*k*_cat,phlorizin_/*K*_m,phlorizin_) × (*k*_cat,xylose_/*K*_m,xylose_)^−1^, the value of 24 × 10^6^ for *Ka*POx_xal shows that phlorizin is by far the preferred substrate. This indicates a distinct transition in substrate selectivity from monosaccharides to glycosides when mutating the wild‐type dimer to an active monomer.

**Table 1:** Apparent steady‐state parameters for dimeric *Ka*POx and monomeric *Ka*POx_xal and *Ka*POx_xalh measured at 30 °C in 50 mm Tris/HCl, pH = 7.5. The values labeled with the asterisk were taken from previously published literature [12]. The abbreviation ‘n.d.’ (not detectable) denotes the absence of any detected activity. The symbol ‘>’ signifies parameters surpassing the observed value.

	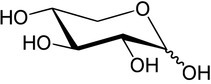 d‐xylose			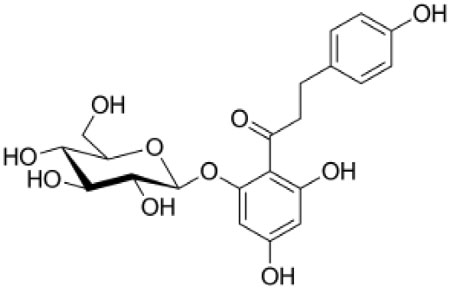 Phlorizin		
	*k*_cat_/s^−1^	*K*_m_/mm	*k*_cat_/*K*_m_/m ^−1^·s^−1^	*k*_cat_/s^−1^	*K*_m_/mm	*k*_cat_/*K*_m_/m ^−1^·s^−1^
*Ka*POx	6.8 ± 0.3*	32 ± 4*	210*	n.d.	n.d.	n.d.
*Ka*POx_xal	0.0013 ± 0.0001	400 ± 80	0.0031	1.2 ± 0.1	0.015 ± 0.002	8 × 10^4^
*Ka*POx_xalh	> 0.00034	> 800	–	0.92 ± 0.05	0.019 ± 0.003	5 × 10^4^

### Dimerization of *Ka*POx depends on hydrophobic effect

**Fig. 3:**
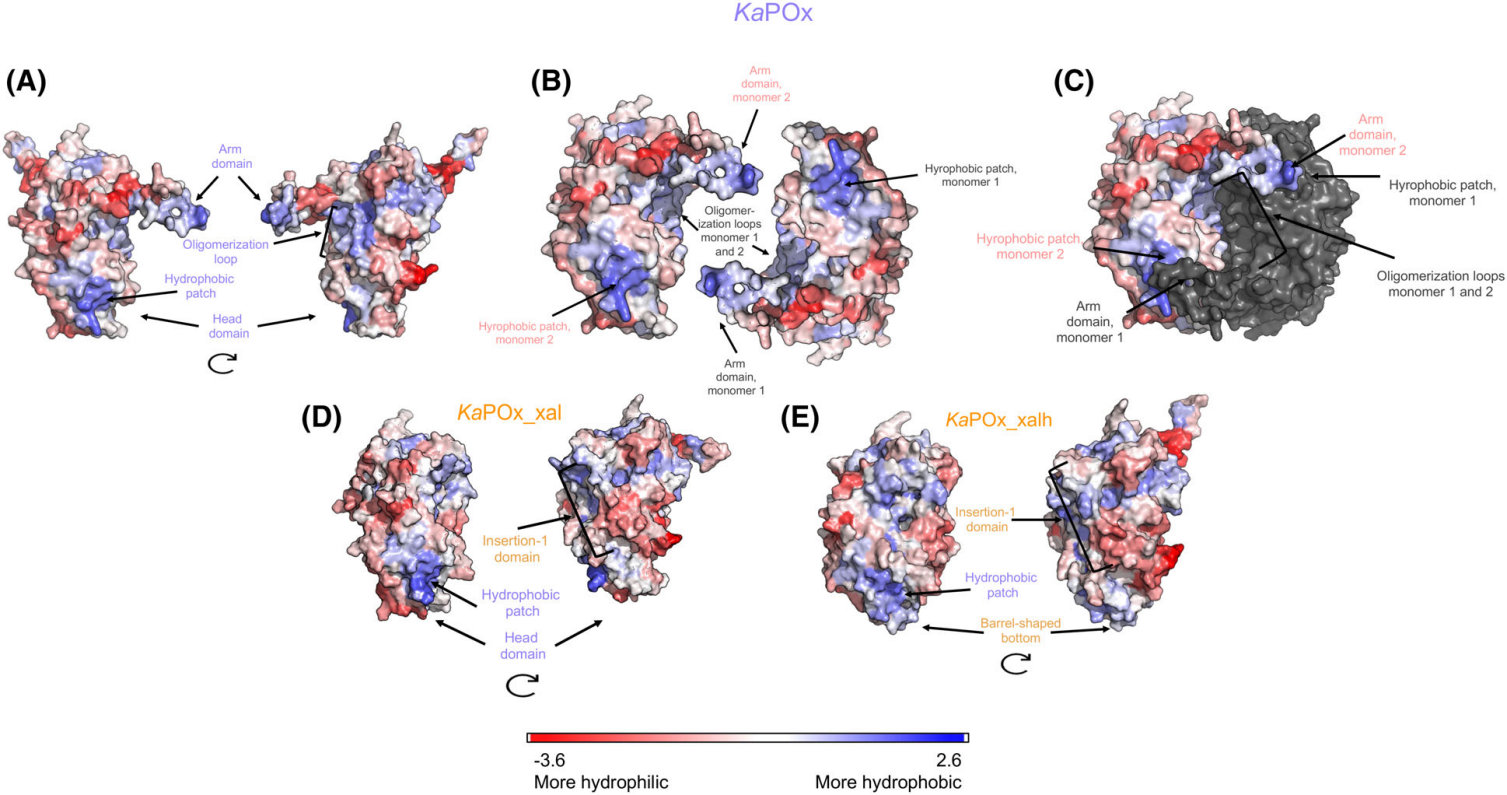
Surface representation of structural models for dimeric *Ka*POx and monomeric *Ka*POx_xal and *Ka*POx_xalh. (A) Representation of wild‐type *Ka*POx as a monomer, highlighting important regions for dimerization. (B, C) Dimeric representation of wild‐type *Ka*POx, showing the attachment of the arm domain of monomer 1 to the hydrophobic patch of monomer 2 and vice versa, and the interaction of the oligomerization loops. (D) Monomeric representation of *Ka*POx_xal, with the arm domain and the oligomerization loop replaced by the more hydrophilic insertion‐1 domain of *Sc*POx. (E) Monomeric representation of *KaPOx*_xalh, where in addition to the replacement with insertion‐1 domain, the head domain is replaced with the barrel‐shaped bottom of *Sc*POx.

To explain the observed behavior of *Ka*POx and its variants, we created models of wild‐type *Ka*POx and the variants *Ka*POx_xal and *Ka*POx_xalh using rosettafold and alphafold 2 [22, 23, 24]. We calculated hydrophobicity scores and then displayed these scores on our models as a heat map (Fig. 3). The wild‐type *Ka*POx monomer has three main hydrophobic surfaces forming a total of two important interactions that play a role in its dimerization (Fig. 3A,B). The hydrophobic arm domain of one monomer interacts with a hydrophobic patch of the second monomer. This patch is formed by the first seven amino acids of the head domain. Furthermore, the oligomerization loop is also hydrophobic, and interacts with the same region of the other monomer when forming a homodimer, burying this surface. In *Ka*POx_xal and *Ka*POx_xalh these interactions are abolished (Fig. 3C,D). The first interaction cannot form when the arm domain is missing, even though the hydrophobic patch in the head domain is still present in *Ka*POx_xal. Furthermore, the head domain is missing in *Ka*POx_xalh, and the patch also becomes slightly less hydrophobic. Exchanging the oligomerization loop to the insertion‐1 domain causes this region to become less hydrophobic. Taken together, the exchanged regions in the variants contribute significantly to hydrophobic effect between two monomers of *Ka*POx.

With respect to the unsuccessful expression of *Sc*POx_al and *Sc*POx_alh, we found a possible explanation in structural models (Fig. S7). The models of the *Sc*POx_al and *Sc*POx_alh variants show that the arm domain is not in the conformation necessary for oligomerization while the addition of the arm domain, the oligomerization loop, and the head domain of *Ka*POx introduces new hydrophobic surfaces. This could explain why the enzyme cannot be expressed in a soluble form in *E. coli* but forms inclusion bodies.

### The orientation of glucose‐binding residues influences glucose specificity in *Ka*POx and its variant *Ka*POx_xalh

**Fig. 4:**
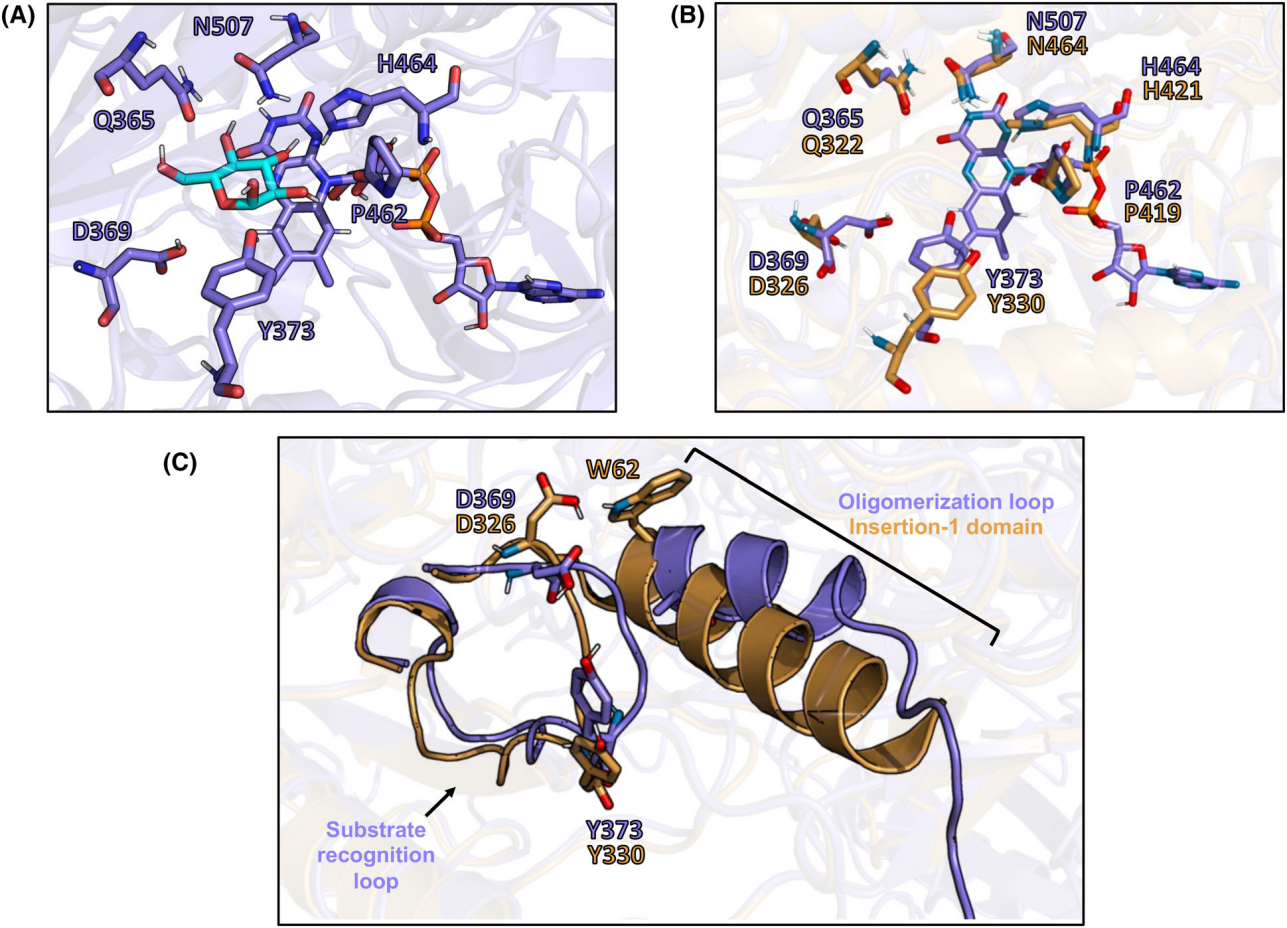
Docking of glucose into the active site of dimeric *Ka*POx and monomeric *Ka*POx_xalh. (A) Docking of d‐glucose into the active‐site pocket of *Ka*POx. The proposed substrate‐binding residues are shown as sticks. The glucose molecule is represented in light blue, and the FAD cofactor is shown as purple sticks. (B) Comparison of the positions of glucose‐binding residues in the alphafold models of *Ka*POx (purple) and *Ka*POx_xalh (golden). The most prominent change in positions is for D369 and Y373 (*Ka*POx numbering). (C) Comparison of the position of the oligomerization loop (*Ka*POx, purple) and insertion‐1 domain (*Ka*POx_xalh, golden). As an example, the interaction of W62 (*Ka*POx_xalh) with D326 (*Ka*POx_xalh) is shown, representing how the change from oligomerization loop to insertion‐1 domain changes the position of the proposed glucose‐binding residues.

As the behavior of *Ka*POx_xal and *Ka*POx_xalh were very similar (Fig. 2 and Table 1), we chose to work with *Ka*POx_xalh as an example of the monomerized *Ka*POx variant. To find possible reasons for the difference in substrate preference between wild‐type *Ka*POx and its variants, we first docked d‐glucose as a reference monosaccharide to the active site of the enzyme. Wild‐type *Ka*POx is highly active with glucose and *Ka*POx_xalh shows little to no activity with the same substrate. There is a clear difference in the glucose binding scores of *Ka*POx and the *Ka*POx_xalh variant (data in online repository). The best orientation of *Ka*POx_xalh has a binding score of −3.1 kcal·mol^−1^, while for wild‐type *Ka*POx the binding scores of the two monomers are −4.9 and −5.2 kcal·mol^−1^ respectively. Glucose‐binding residues are highly conserved in known fungal POxs [13, 25]. These residues are also conserved in *Ka*POx and the modified variants (Table S4), except for A546 (*Phanerochaete chrysosporium* POx (*Pc*POx) numbering), which is replaced by a proline in *Ka*POx. However, as this residue only interacts via its backbone carbonyl oxygen with 3‐fluoro‐D‐glucose in the *Pc*POx X‐ray structure (PDB 4MIG) [25], this replacement should not influence glucose binding, and wild‐type *Ka*POx is highly active with d‐glucose. Indeed, glucose seems to bind to these residues via hydrogen bonds in the docked structures (Fig. 4A). The main difference between wild‐type and its variant *Ka*POx_xalh is near the putative glucose‐binding residues, in the orientation of the substrate recognition loop [26]. The amino acid region T367‐L384 (T324‐L341 in *Ka*POx_xalh, Fig. 4B and Fig. S1), which contains the glucose‐binding residues D369 and Y373, is also close enough to influence the position of Q365 found in an adjacent β‐sheet. While this loop was not mutated, it is spatially close to the oligomerization domain, which was exchanged for the insertion‐1 domain. The exchange of A62 in *Ka*POx to W62 in *Ka*POx_xalh causes a new interaction, as the side chain of W62 is directed towards the loop (Fig. 4C), so that it can interact with D369, modifying its position. This also modifies the position of side chains in the entire loop, including the position of Y373 (*Ka*POx numbering). Furthermore, the position of the entire insertion‐1 domain shifts in *Ka*POx_xalh, which can cause further changes in the active site architecture.

We acknowledge that the positions of flexible loops can be unreliable in models, and the alphafold structures have a pLDDT score below 90 in these regions in both models of *Ka*POx and *Ka*POx_xalh (Fig. S8), indicating that the predicted positions of the side chains in this region are not highly reliable. However, they explain the experimental observation of significantly increased *K*_m_ values of *Ka*POx_xalh towards monosaccharides (Table 1). Furthermore, the exchange of the small, hydrophobic A62 to the bulky W62 will certainly have structural effects on the substrate recognition loop—even if the modification is not to the exact positions predicted here.

### Glycoside reactivity is likely influenced by steric hindrance

To elucidate why *Ka*POx shows a significantly lower activity towards glycosides, we docked the *O*‐glycoside phlorizin into the *Ka*POx and *Ka*POx_xalh models. To do so, we had to remove a short section in the gating segment (R368‐H372 in *Ka*POx and R325‐H329 in *Ka*POx_xalh) in the structural models of the proteins, as it was in a closed position in the original alphafold models, and it did not allow for binding poses with the larger sized aglycone attached to the sugar moiety.

**Fig. 5:**
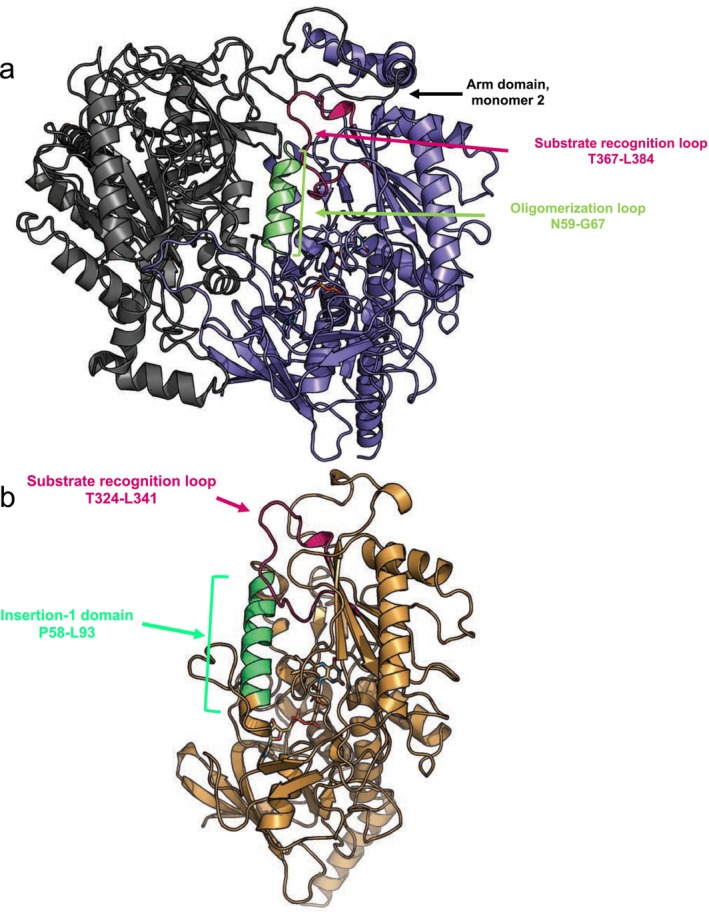
The accessibility of the active site for the glycoside phlorizin of dimeric *Ka*POx and monomeric *Ka*POx_xalh. (A) Structural prediction of the *Ka*POx dimer. The substrate recognition loop of the first monomer (pink) is spatially close to the arm domain of the second monomer (black). The oligomerization loop is shown is light green. (B) *Ka*POx_xalh monomer. No steric hindrance from the arm domain of the other monomer or oligomerization loop is obvious in this case. [Correction added on 12 February 2025 after first online publication: Figure 5 and its legend have been replaced in this version as an incorrect figure and legend had been inadvertently compiled into the article.]

In this case, the docking scores of *Ka*POx (−8.3 and −8.1 kcal·mol^−1^) are similar that of *Ka*POx_xalh (−7.8). While these scores can be unreliable due to the missing amino acids, the removed section only contains one of the proposed substrate recognition residues (D369). Yet, *Ka*POx shows no activity for this substrate, while *Ka*POx_xalh does. This indicates that the lack of activity with glycosides in *Ka*POx is not caused by substrate binding itself, but by the accessibility of the active site. The active‐site pocket is surrounded by the substrate recognition loop and the α‐helix of the oligomerization or insertion‐1 domain (Fig. 5). While in wild‐type *K*aPOx the mobility of the oligomerization loop is hindered by the same amino acid region of the second monomer, and the substrate recognition loop is directly surrounded by the arm domain of the second monomer (Fig. 5A), these hindrances are not present in the monomeric *Ka*POx_xalh, which allows this monomeric variant to accommodate substrates with a bulky aglycone part into their active site pocket (Fig. 5B).

## Discussion

Here we report for the first time the engineering of the oligomeric state of POxs and CGOx to alter their substrate scope. Deletion of the oligomerization loop and the arm domain as in the variant *Ka*POx_xal resulted in a functional monomeric enzyme with kinetic properties showing a high preference towards glycosides rather than monosaccharides. Similarly, *Ka*POx_xalh shows similar kinetic properties despite additionally deleting the head domain (Fig. 2, Table 1). When comparing the kinetic parameters of *Ka*POx_xal and *Ka*POx_xalh to the naturally occurring wild‐type monomeric *C*‐glycoside oxidases from *Rhizobium* sp. GIN611 (GlycDH), *Sc*POx, *Ps*POx and *Mt*CArA, all sharing the same sequence space, we note that the engineered variants display the same preference for glycosides over monosaccharides, thus behaving like CGOx rather than POx [21].

These experimental results were confirmed by modeling. We found that the dimerization of *Ka*POx is governed by hydrophobic effect surfaces as indicated by structural models. When removing or exchanging the regions in *Ka*POx that provide these hydrophobic surfaces, the enzyme becomes a monomer (Fig. 3). This is in line with previous observations on oligomerization [4, 6, 27]. Fungal POxs are known to be tetrameric, forming a dimer of dimers, where the subunit‐subunit interactions are governed by a large number of hydrogen bonds as well as hydrophobic contacts as shown by several X‐ray structures. The residues that provide interactions for oligomerization between subunits are mainly located in the oligomerization loop and the arm domain [20]. The head domain is also presumed to contribute to oligomerization; however, its exact role is yet unclear. Its surface in wild‐type *Ka*POx is hydrophilic, which may explain why this enzyme does not form tetramers and why fungal POxs have been predominantly reported as tetrameric structures (Fig. S9).

Differences in binding monosaccharides and glycosides can be explained by structural models of wild‐type *Ka*POx and its variant *Ka*POx_xalh. The amino acid composition of the substrate loop differs considerably between monomeric and multimeric POxs (Fig. S1, Table S1). While this loop was not mutated in our monomerized *Ka*POx variants, substrate specificity was affected significantly. This is most likely a steric effect due to the mutations in spatially close regions [4, 28]. These regions have a low confidence score in our *Ka*POx and *Sc*POx models (Figs S7 and S8), with low‐scoring regions indicating highly flexible or disordered regions [29]. We propose that exchanging this loop in addition to the introduced monomerization could further push the enzyme towards preferring glycosides instead of monosaccharides as substrates. This loop exchange by itself would likely not be sufficient to swap substrate preference without the monomerization of the enzyme though, as the glycoside preference is dependent on the steric accessibility of the active site, which is greatly affected by the oligomerization.

In general, few mechanisms to turn monomeric enzymes into dimers have been described, namely domain swapping, small‐scale conformational changes, point mutations, introduction of stabilizing ligands and ions on interfaces, of posttranslational modifications such as phosphorylation and disulphide bond formation, and of insertions/deletions [6]. It was also shown that insertions/deletions tend to be located on the interaction surface, where even a small change due to these indels can have a drastic change on protein complex stability and specificity. Intriguingly, protein multimerization interfaces are somewhat more conserved than the protein surface itself, but, at the same time, less than the protein core; they tend to evolve at a slow rate [30]. Loops and strands occur more frequently in regions enabling dimerization and on the protein surface [6]. These observations are indeed also seen in the POx/CGOx sequence space. For example, already reported insertions and deletions in the *pox/cgox* genes such as the insertion‐1 domain in *Ps*POx seem to play a crucial role in the preference to form monomers [14]. A study on glycosyltransferases by Hashimoto *et al*. reports a similar observation in which the oligomeric state of glycosyltransferases influences their activity [31, 32].

## Conclusion

Interactions between plants and microbes are based on intricate networks of various signals and chemicals. Among many secondary metabolites produced by plants, glycosides are antimicrobial molecules that protect plants against microbial invasion. Soil‐thriving bacteria express genes encoding *C*‐glycoside oxidase and *C*‐glycoside deglycosylase to degrade glycosides [17, 33], thus detoxifying these antimicrobial compounds. In parallel to this, genes encoding pyranose oxidase can be found in the genome of certain plant and soil‐associated microbes, putatively involved in the depolymerization of plant cell wall components such as lignin. We propose that monomeric *C*‐glycoside deglycosylases first evolved for the cleavage of the glycosidic bond in a number of plant‐derived glycosides and later evolved into dimeric bacterial enzymes that play a role in lignocellulose deconstruction by providing hydrogen peroxide to peroxidases.

## Materials and methods

### Design of mutants

Based on our previous work, we selected dimeric *Ka*POx (UniProt A0A1E7NAU4) and monomeric *Sc*POx (UniProt A0A117Q443) [11, 12] for further studies. A multiple sequence alignment was performed by mafft (v. 7.0.26) using the method FFT‐NS‐I [34]. The initial structural predications of the *Ka*POx and *Sc*POx monomers were made with rosettafold (https://robetta.bakerlab.org/submit.php, accessed in August 2023) [24]. Structural alignments of *Ka*POx and *Sc*POx in pymol (v. 2.5.2; educational license; Schrödinger, New York, NY, USA) assisted in determining the arm and head domain regions as well as the insertion‐1 segment and the barrel‐shape bottom, which was used to design variants. X‐ray crystal structures of *Ps*POx (PDB 7QF8, 7QFD, 7QVA) and *Mt*CarA (PDB 7DVE) were used for structural alignments [10, 14]. Structural predictions (Fig. 1) were visualized with the software ucsf chimerax (v. 1.6.1; academic license) [35]. Alignments were visualized by bioedit (v. 7.2; Thomas A. Hall).

### Synthesis and cloning of variants

Nucleotide sequences were synthesized and cloned into the vector pET‐21a(+) between the restriction sites *Nde*I and *Hind*III by a commercial clonal gene service (BioCat, Heidelberg, Germany). Coding sequences were cloned in a way that they contain a C‐terminal His_6_ tag. These constructs also carry the ampicillin‐resistance marker cassette. Molecular properties were calculated by ProtParam (accessed in January 2024) [36].

For re‐cloning of the *scpox_al* and *scpox_alh* genes, HiFi DNA assembly was used (New England Biolabs, Ipswich, MA, USA). Primers were designed by nebuilder (v. 2.9.1; https://nebuilder.neb.com/#!/, accessed in October 2023). The list of primers used is given in Table S5. HiFi DNA assembly was done in accordance to the corresponding New England Biolabs manual. Destination plasmids for reconstructing were pD441 (modified to include an N‐terminal His_6_ tag, PreScission cleavage site and multicloning site) and pNIC‐CTHO.

### Overexpression and protein purification

Calcium‐competent *Escherichia coli* T7 Express cells (New England Biolabs) were transformed using the heat‐shock transformation procedure. For re‐cloning of *Sc*POx variants, *E. coli* strains BL21(DE3), Lemo21(DE3) (New England Biolabs), C41(DE3) and C43(DE3) (Sigma‐Aldrich, St. Louis, MO, USA) were transformed using the same protocol. In addition, all *E. coli* cell lines were also co‐transformed with the plasmid pG‐KJE8 (TaKaRa, Shiga, Japan) containing the genes for the chaperones dnaK‐dnaJ‐grpE and groES‐groEL.

Gene expression was induced with 500 μm isopropyl β‐d‐1‐thiogalactopyranoside (IPTG; Lactan, Graz, Austria) when cell culture density OD_600_ reached 0.6–1. Overexpression lasted for 18 h at 20 °C. Cells were grown in Luria‐Bertani medium with ampicillin (100 μg·mL^−1^) (all components from Carl Roth, Karlsruhe, Germany). Collection of the cell pellet was done by centrifugation of cell cultures for 20 min at 9000 ***g*** at 8 °C (centrifuge Avanti J‐26 XP, rotor JA‐10; Beckman Coulter, Brea, CA, USA).

Protein purification was initiated by sonication of the resuspended cell pellet using an ultrasonic homogenizer (Sonopuls; Bandelin, Berlin, Germany) at 120 V and 50% cycle for 10 min, thrice repeated with 5 min breaks. Cells were resuspended in 50 mm Tris/HCl, pH = 7.5 supplemented with 10 mm FAD, 10 mg·mL^−1^ lysozyme, 10 U DNaseI and 2 mm MgCl_2_ (all additives from Sigma‐Aldrich). The soluble fraction was separated from cell debris by centrifugation for 1 h at 60 000 ***g*** at 4 °C (centrifuge Avanti J‐26 XP, rotor JA‐25.50). Proteins were purified by immobilized metal affinity chromatography (IMAC) using the ÄKTA Go chromatography system (Cytiva, Marlborough, MA, USA). A His‐Trap column of 5 mL (Cytiva) was equilibrated with purification buffer (150 mm NaCl, 5% glycerol and 50 mm Tris/HCl, pH = 7.5) containing 30 mm imidazole. Protein elution was performed with a linear gradient (0–100%, 10 min, 1 mL·min^−1^) of purification buffer containing 500 mm imidazole. An additional polishing step was used for the variant *Ka*POx_xalh by size‐exclusion chromatography (SEC). A column of 120 mL packed with Superdex 75 (Cytiva, in‐house packed column) was equilibrated with polishing buffer (150 mm NaCl, 50 mm Tris/HCl, pH = 7.5). Protein elution was done with the same buffer and a flow rate of 1 mL·min^−1^. The selected fractions were pooled, desalted and concentrated using ultraconcentrators (molecular weight cut‐off 30 kDa; Merck Millipore, Billerica, MA, USA). Purified proteins were stored at −80 °C in storage buffer (150 mm NaCl, 10% glycerol and 50 mm Tris/HCl, pH = 7.5). All buffer components were from Carl Roth. Purified *Ka*POx protein was prepared as reported before [12].

To check the purity of fractions and protein samples, sodium dodecyl sulfate–polyacrylamide gel electrophoresis (SDS/PAGE) was done as previously described [11].

### Spectral measurements; determination of oligomeric state; thermostability measurements

Spectra of protein samples were recorded between 200 and 500 nm with a diode array spectrophotometer at room temperature (8453 UV–visible spectroscopy system, Agilent, Santa Clara, CA, USA). FAD occupancy was calculated using the absorbance of the protein samples at 450 nm and the extinction coefficient of free FAD in solution (ε_FAD,450nm_ 11 300 m ^−1^·cm^−1^), which, based on Beer‐Lamber law, yielded in concentration of protein loaded with FAD. The ratio of protein loaded with FAD divided by the total protein concentration of our enzyme samples gave the percentage of protein loaded with FAD. Protein concentrations were determined using the absorbance at 280 nm and the calculated extinction coefficients of the protein variants (ε_*Ka*POx_xal,280nm_ 64 985 m ^−1^·cm^−1^, ε_*Ka*POx_xalh,280nm_ 64 985 m ^−1^·cm^−1^).

Size‐exclusion chromatography‐light scattering (SEC‐LS) was used to characterize the recombinant proteins in solutions with respect to their purity, oligomerization or aggregate formation, as well as molecular masses. Analyses were performed on an OMNISEC multi‐detector gel permeation chromatography/size‐exclusion chromatography system (GPC/SEC) equipped with a refractive index detector, a right‐angle light scattering detector, a low‐angle light scattering detector and a UV/Vis photodiode array detector (Malvern Panalytical; Malvern UK). A Superdex 200 Increase 10/300 GL column (Cytiva) was used and equilibrated with Dulbecco's phosphate buffered saline (without Ca^2+^ and Mg^2+^) (PAN‐Biotech, Aidenbach, Germany) as running buffer. Experiments were performed at a flow rate of 0.5 mL·min^−1^ at 25 °C and analyzed using the omnisec software (v. 11.40; Malvern Panalytical, UK). Proper performance of the instrument was ensured by calibration and verification using 200 μg Pierce bovine serum albumin standard (ThermoFisher Scientific, Waltham, MA, USA). Prior to analysis, samples were centrifuged (16 000 ***g***, 10 min), and filtered through 0.1‐μm Durapore polyvinylidene fluoride centrifugal filters (Merck Millipore). A volume of 100 μL of each sample, having protein concentrations between 1.2 and 1.6 mg·mL^−1^, was injected. To evaluate whether protein concentrations affect the oligomerization behavior, samples with protein concentrations of 4.7 and 11.1 mg·mL^−1^, were studied as well.

Thermostability of proteins was evaluated using the Thermo FAD assay as previously described [37]. For these purposes, a real‐time PCR cycler (iCycler; Bio‐Rad, Hercules, CA, USA) was set to record the increase in the fluorescence signal of the FAD cofactor upon unfolding of the protein. The fluorescent signal was detected using the SYBR‐green filter (mMyiQ detection system; Bio‐Rad). Protein samples (in 25 μL) were prepared in a concentration of 2 mg·mL^−1^. Recordings were performed over a gradient of 25 to 80 °C with increments of 1°·min^−1^ in triplicates.

### Screening for activity; steady‐state parameters determination

The following substrates were used for an initial screening for activity: the monosaccharides d‐glucose and d‐xylose (Carl Roth), the *C*‐glycosides carminic acid (Glentham Life Sciences, Corsham, UK), isoorientin (homoorientin) (abcr, Karlsruhe, Germany), isovitexin (TargetMol, Boston, MA, USA), mangiferin (Sigma‐Aldrich), puerarin (abcr), the *O*‐glycosides fraxin (BLDpharm, Puding, China), phlorizin (Sigma‐Aldrich), salicin, rutin (all from abcr), and the *S*‐glycoside sinigrin (Sigma‐Aldrich). Stock solutions of monosaccharides were prepared in water, and those of glycosides in pure dimethyl sulfoxide (DMSO). The activity was assessed employing the 2,6‐dichlorophenolindophenol (DCIP) or 10‐acetyl‐3,7‐dihydroxyphenoxazine (Chemodex, St. Gallen, Switzerland) (AmplexRed)‐coupled assay, as previously described [21]. In short, activity measurements were carried out by mixing a suitable amount of enzyme, 7.5 U horseradish peroxidase (Sigma‐Aldrich P8250), 300 μm of DCIP or 50 μm of AmplexRed, and 1 μL of substrate in 50 mm Tris/HCl (pH 7.5) to a final volume of 0.1 mL. To ensure no background activity was detected as false positive, blank reactions without protein and without substrate were measured as well. Change in absorbance was followed at 520 and 560 nm for the DCIP and AmplexRed‐coupled assay, respectively, while activity was calculated using the corresponding extinction coefficients (ε_DCIP,520nm_ 6.6 mm ^−1^·cm^−1^, ε_AmplexRed,560nm_ 54.0 mm ^−1^·cm^−1^). The measurements were performed in triplicates and at 30 °C in 50 mm Tris/HCl, pH = 7.5. The activity data for *Ka*POx were taken from the original publications [12, 21], and only activity with glycosides was assessed here. Concentration during substrate screening was 6 mg·mL^−1^ for *Ka*POx, 2.5 mg·mL^−1^ for *Ka*POx_xal and 1.2 mg·mL^−1^ for *Ka*POx_xalh. Enzymatic reactions were performed in 200 μL volumes, and the absorbance was measured spectrophotometrically (EnSpire plate reader; PerkinElmer, Waltham, MA, USA). Heatmaps illustrating the results from screening for activity were made by the Python data visualization library Seaborn [38].

Apparent steady‐state kinetic parameters were assessed by the AmplexRed‐coupled assay. The concentration of the initial electron acceptor was kept constant (air O_2_~250 μm), while the concentration of electron donors (d‐xylose, phlorizin) was varied. The concentration range for d‐xylose was 0–800 mm, and that for phlorizin was 0–0.3 mm. Concentration of *Ka*POx_xal was 0.12 mg·mL^−1^ in the assay with phlorizin and 2.5 mg·mL^−1^ in the assay with d‐xylose, while concentration of *Ka*POx_xalh was 0.31 mg·mL^−1^ in the assay with phlorizin and 1.2 mg·mL^−1^ in the assay with d‐xylose. All curves were plotted and fitted to the Michaelis–Menten curve (non‐linear regression, equation category ligand binding, one site saturation) in sigmaplot (v.14.0; Systat Software, Düsseldorf, Germany). *k*_cat_ values were calculated using the *v*_max_ values and the theoretical molecular mass of each enzyme (*M*_r,*Ka*POx_xal_ = 58.7 kDa, *M*_r,*Ka*POx_xalh_ = 56.9 kDa).

### Modeling hydrophobicity

We used the alphafold structure of *Ka*POx available in the alphafold Protein Structure database [22, 23]. We used an in‐house alphafold 2 server to build structural models of *Ka*POx_xal and *Ka*POx_xalh, and used models created with rosettafold (https://robetta.bakerlab.org/submit.php, accessed in August 2023) [24] for *Sc*POx, *Sc*POx_al, and *Sc*POx_alh. For *Pc*POx and *Tm*POx, the corresponding crystal structures were used (PDB 4MIG and 1TT0) [20, 25]. A hydrophobicity score was calculated for each amino acid in the variants using the Expasy server ProtScale [39], with the algorithm by Kyte and Doolittle [40], taking a window size of 9 and a linear weight variation model. The obtained scores were mapped as b‐factors to the models, using pymol, with a red‐white‐blue color scale going from −3.6 (hydrophilic) to 2.6 (hydrophobic), and represented in a surface model for analysis.

### Docking glucose and phlorizin, structural analysis

To create a model of the *Ka*POx dimer, the apo alphafold structure was duplicated in pymol, and the two monomers were aligned to the two corresponding monomers of fungal *Tm*POx (PDB 1TT0) [20]. The FAD molecules were also fitted from the aligned 1TT0 structure. The holo homodimer was then energy‐minimized in gromos [41] in vacuum, using the steepest descent algorithm and the 54a8 parameter set. For modeling holo *Ka*POx_xalh, we first energy‐minimized the apo structure as above, then aligned the *Pc*POx X‐ray structure (PDB: 4MIG) [25] to it, and after alignment, fitted the FAD from the X‐ray structure. Then, holo *Ka*POx_xalh was energy‐minimized once more. The structures were converted to PDBQT format using openbabel [42], atom types for the FAD molecules were defined by hand. For obtaining glucose and phlorizin structures for docking, the ideal sdf‐format structures were obtained from the RCSB PDB database [43], and converted to PDBQT format using openbabel.

Docking was done using autodock vina [44, 45]. The central coordinate was defined at the O5 atom of the 3‐deoxy‐3‐fluoro‐glucopyranose from the X‐ray structure of *Pc*POx, after aligning it with the holo *Ka*POx or *Ka*POx_xalh structures. The box size was defined to be 10 × 10 × 10 Å^3^ for docking d‐glucose, and 15 × 15 × 15 Å^3^ for phlorizin. Exhaustiveness was set to 32. For docking phlorizin, amino acids R325‐H329 in *Ka*POx_xalh and R368‐H372 in *Ka*POx were removed from the PDBQT structures. The obtained docking scores correspond to a predicted affinity of binding of the small molecule, in kcal·mol^−1^, therefore lower scores correspond to stronger binding. Structures were analyzed and figures rendered (Figs 3, 4, 5 and Figs S7–S9) using pymol.

## Conflict of interest

The authors declare no conflict of interest.

## Author contributions

AK: Data curation (lead); formal analysis (lead); methodology (equal); visualization (lead); writing – original draft preparation (lead); writing – review and editing (equal). EH: Data curation (equal); formal analysis (equal); methodology (equal); visualization (equal); writing – review and editing (equal). CP: Conceptualization (equal); funding acquisition (supporting); resources (supporting); supervision (supporting); writing – review and editing (equal). CO: Conceptualization (equal); funding acquisition (supporting); resources (supporting); supervision (supporting); writing – review and editing (equal). DH: Conceptualization (lead); funding acquisition (lead); project administration (lead); resources (lead); supervision (lead); writing – review and editing (equal).

### Peer review

The peer review history for this article is available at https://www.webofscience.com/api/gateway/wos/peer‐review/10.1111/febs.70004.

## Supporting information


**Fig. S1.** Multiple sequence alignment of the bacterial dimeric *Ka*POx, monomeric *Sc*POx, *Ps*POx and *Mt*CarA and fungal tetrameric *Tm*POx.
**Fig. S2.** SDS/PAGE of purified *Ka*POx_xal and *Ka*POx_xalh preparations.
**Fig. S3.** SEC‐LS chromatograms of purified *Ka*POx_xal and *Ka*POx_xalh.
**Fig. S4.** SEC‐LS chromatograms of a KaPOx_xalh sample after purification in different concentrations.
**Fig. S5.** UV/Vis absorption spectra (300–500 nm) of *Ka*POx_xal and *Ka*POx_xalh protein samples.
**Fig. S6.** Michaelis–Menten curves for variants *Ka*POx_xal and *Ka*POx_xalh.
**Fig. S7.** Error estimates in Å for each amino acid in the RoseTTAFold models of *Sc*POx and its variants.
**Fig. S8.** alphafold2 models of *Ka*POx and its variants, colored from red to blue according to the pLDDT (reliability score) assigned to each amino acid, and pLDDT scores plotted against the amino acid numbers for each variant.
**Fig. S9.** Comparison of the hydrophobic tetramerization surfaces of *Tm*POx (PDB 1TT0) and a wild‐type *Ka*POx model.
**Table S1.** Amino acid sequences of the wild‐type enzymes *Ka*POx and *Sc*POx, and variants *Ka*POx_xal, *Ka*POx_xalh, *Sc*POx_al. and *Sc*POx_alh.
**Table S2.** Biochemical properties of wild‐type *Ka*POx and its variants *Ka*POx_xal and *Ka*POx_xalh.
**Table S3.** Substrates for which an activity was identified during the initial screening.
**Table S4.** Putative glucose‐binding residues in *Ka*POx and the *Ka*POx_xalh variant.
**Table S5.** List of primers used in this study to re‐clone *Sc*POx_al and *Sc*POx_alh.

## Data Availability

All models, hydrophobicity scores and docking results are available at the repository https://doi.org/10.5281/zenodo.10782082. All other data are available upon request from the authors.
