# Supplementary material for: Shifting the substrate scope of dimeric pyranose oxidase from monosaccharide to glycoside preference through oligomeric state modification
Source: FEBS J. 2025 Feb 6;292(9):2323–37. doi: 10.1111/febs.70004 (PMC12062775; doi:10.1111/febs.70004)
Supplement: Supplementary file 1 — Fig. S1. Multiple sequence alignment of the bacterial dimeric KaPOx, monomeric ScPOx, PsPOx and MtCarA and fungal tetrameric TmPOx. Fig. S2. SDS/PAGE of purified KaPOx_xal and KaPOx_xalh preparations. Fig. S3. SEC‐LS chromatograms of purified KaPOx_xal and KaPOx_xalh. Fig. S4. SEC‐LS chromatograms of a KaPOx_xalh sample after purification in different concentrations. Fig. S5. UV/Vis absorption spectra (300–500 nm) of KaPOx_xal and KaPOx_xalh protein samples. Fig. S6. Michaelis–Menten curves for variants KaPOx_xal and KaPOx_xalh. Fig. S7. Error estimates in Å for each amino acid in the RoseTTAFold models of ScPOx and its variants. Fig. S8. alphafold2 models of KaPOx and its variants, colored from red to blue according to the pLDDT (reliability score) assigned to each amino acid, and pLDDT scores plotted against the amino acid numbers for each variant. Fig. S9. Comparison of the hydrophobic tetramerization surfaces of TmPOx (PDB 1TT0) and a wild‐type KaPOx model. Table S1. Amino acid sequences of the wild‐type enzymes KaPOx and ScPOx, and variants KaPOx_xal, KaPOx_xalh, ScPOx_al. and ScPOx_alh. Table S2. Biochemical properties of wild‐type KaPOx and its variants KaPOx_xal and KaPOx_xalh. Table S3. Substrates for which an activity was identified during the initial screening. Table S4. Putative glucose‐binding residues in KaPOx and the KaPOx_xalh variant. Table S5. List of primers used in this study to re‐clone ScPOx_al and ScPOx_alh. [file FEBS-292-2323-s001.pdf]

SUPPLEMENTAL DATA

Shifting the substrate scope of dimeric pyranose oxidase from monosaccharide to glycoside preference through oligomeric state modification

Anja Kostelac[1,2#], Enikő Hermann[1,2,3#], Clemens Peterbauer[1], Chris Oostenbrink[3,4], Dietmar Haltrich[1]

#Shared first authorship

1 Department of Food Science and Technology, BOKU University, Vienna, Austria

2 Doctoral Programme BioToP - Biomolecular Technology of Proteins, BOKU University, Vienna, Austria

3 Department of Material Science and Life Sciences, BOKU University, Vienna, Austria

4 Christian Doppler Laboratory for Molecular Informatics in the Biosciences, BOKU University, Vienna, Austria

Abbreviations:

IMAC, immobilized metal affinity chromatography

*Ka*POx, pyranose oxidase from *Kitasatospora aureofaciens*

*Mt*CarA, FAD-dependent *C*-glycoside 3-oxidase from *Microbacterium trichothecenolyticum*

*Pc*POx, pyranose oxidase from *Phanerochaete chrysosporium*

*Ps*POx, pyranose oxidase from *Pseudarthrobacter siccitolerans*

*Sc*POx, pyranose oxidase from *Streptomyces canus*

SDS-PAGE, sodium dodecyl sulfate–polyacrylamide gel electrophoresis

SEC, size exclusion chromatography

SEC-LS, Size-exclusion chromatography-light scattering

*Tm*POx, pyranose oxidase from *Trametes multicolor*

```
                       10         20         30         40         50         60
                ....|....| ....|....| ....|....| ....|....| ....|....| ....|....|
KaPOx        1  MI-------- ---------- ---------- ---------- ---TRYTDTL VVGSGPVGAT  19
ScPOx        1  MT-------- ---------- ---------- ---------- --HTPRTDVL IVGSGIMGSL  20
KaPOx_xal    1  MI-------- ---------- ---------- ---------- ---TRYTDTL VVGSGPVGAT  19
KaPOx_xalh   1  MI-------- ---------- ---------- ---------- ---TRYTDTL VVGSGPVGAT  19
ScPOx_al     1  MT-------- ---------- ---------- ---------- --HTPRTDVL IVGSGIMGSL  20
ScPOx_alh    1  MT-------- ---------- ---------- ---------- --HTPRTDVL IVGSGIMGSL  20
PsPOx        1  MS-------- ---------- ---------- ---------G HRYPAAVDVA IVGSGPTASA  23
MtCarA       1  MS-------- ---------- ---------- ---------T RVYPAQVDVA IVGSGPAGAT  23
TmPOx        1  MSTSSSDPFF NFAKSSFRSA AAQKASASSL PPLPGPDKKV PGMDIKYDVV IVGSGPIGCT  60

                                                      Oligomerization loop
                       70         80         90        100        110        120
                ....|....| ....|....| ....|....| ....|....| ....|....| ....|....|
KaPOx       20  FARTLVES-- GREVLMVDAG AQLSP-RPGE HLKNAYIYQH NTNLFASIIR GHLHLLSVPT  76
ScPOx       21  VARLLRRSDP ALHITMADGG SPIGG-VPGR HLHDL----- D--------- ----------  55
KaPOx_xal   20  FARTLVES-- GREVLMVDAG AQLSP-RPGE HLKNAYIYQH NT-------- ----------  58
KaPOx_xalh  20  FARTLVES-- GREVLMVDAG AQLSP-RPGE HLKNAYIYQH NT-------- ----------  58
ScPOx_al    21  VARLLRRSDP ALHITMADGG SPIGG-VPGR HLHDL----- DDNLFASIIR -HLHLLSVPT  73
ScPOx_alh   21  VARLLRRSDP ALHITMADGG SPIGG-VPGR HLHDL----- DDNLFASIIR -HLHLLSVPT  73
PsPOx       24  YARILSEEAP GATIAMFEVG PTVSN-PPGA HVKNI--EDP DS-------R SLAQRASEGP  73
MtCarA      24  YARILSERAS SATIAMFEVG PTVSD-PPGA HVKNI--ADA DE-------R AHAQRRSEGP  73
TmPOx       61  YARELVGA-- GYKVAMFDIG EIDSGLKIGA HKKNTVEYQK NIDKFVNVIQ GQLMSVSVPV 118

Arm domain
                   Insertion-1 domain           Insertion-1 domain
       (corresponding to oligomerization loop)  (corresponding to arm domain)
                      130        140        150        160        170        180
                ....|....| ....|....| ....|....| ....|....| ....|....| ....|....|
KaPOx       77  SARAELAVDP AAMAELGSNR SSARNAENPD QDPYRNLSAA AACYA----- ---------- 121
ScPOx       55  --------DP DLWSRYNEKV ATGIQGMYTG AEVVRDVAGS LPDITPGMFH ALAFGEDAEA 107
KaPOx_xal   58  ---------P DLWSRYNEKV ATGIQGMYTG AEVVRDVAGS LPDLA----- ----------  94
KaPOx_xalh  58  ---------P DLWSRYNEKV ATGIQGMYTG AEVVRDVAGS LPDLA----- ----------  94
ScPOx_al    74  SARAELAVDP AAMAELGSNR SSARNAENPD QDPYRNLSAA AACYTPGMFH ALAFGEDAEA 133
ScPOx_alh   74  SARAELAVDP AAMAELGSNR SSARNAENPD QDPYRNLSAA AACYTPGMFH ALAFGEDAEA 133
PsPOx       74  GAGAA---TV NSPGAVKSGE RRARPGTYLL QDGYA----- ---------- --FPGED--G 111
MtCarA      74  HARED---DD RVGGIVKSAQ RRARPGTYLL ESGYQ----- ---------- --ADGED--G 111
TmPOx      119  NT---LVVDT LSPTSWQAST FFVRNGSNPE QDPLRNLSGQ AVTRV----- ---------- 160

                      190        200        210        220        230        240
                ....|....| ....|....| ....|....| ....|....| ....|....| ....|....|
KaPOx      121  ---------V GGMGTHWTGA TPR----HHP VLERYDGISD Q-EWDGLYGE AERLLRVSAR 167
ScPOx      108  MPQAALAWNA GGMGVHWTAA TPW----PAG D-EVFDFGDP D-AWAADLDT ARRLLAVTPA 161
KaPOx_xal   94  ---------V GGMGTHWTGA TPR----HHP VLERYDGISD Q-EWDGLYGE AERLLRVSAR 140
KaPOx_xalh  94  ---------V GGMGTHWTGA TPR----HHP VLERYDGISD Q-EWDGLYGE AERLLRVSAR 140
ScPOx_al   134  MPQAALAWNA GGMGVHWTAA TPW----PAG D-EVFDFGDP D-AWAADLDT ARRLLAVTPA 187
ScPOx_alh  134  MPQAALAWNA GGMGVHWTAA TPW----PAG D-EVFDFGDP D-AWAADLDT ARRLLAVTPA 187
PsPOx      112  MPVAAMSSNV GGMAAHWTAA CPR----PGG K-ERIPFLP- --DLEELLND ADRLLGVTTH 163
MtCarA     112  LPVAAFSSNV GGMAAHWTGA CPR----PND S-ERIGFLDE TGELDELLSE GERLLGVTTD 166
TmPOx      160  ---------V GGMSTHWTCA TPRFDREQRP LLVKDDADAD DAEWDRLYTK AESYFQTGTD 211

                      250        260        270        280        290        300
                ....|....| ....|....| ....|....| ....|....| ....|....| ....|....|
KaPOx      168  EFDFSIRQHL VTEALRREFS EL-PDGYQVQ SLPLAARRRR DNPRMVHWTG VDTVLG---- 222
ScPOx      162  PIGPTKVGEL VLDVLRRRYG GTGPADRAPQ PMPMAVTPTP SGP--MPRTA PGTIFP---- 215
KaPOx_xal  141  EFDFSIRQHL VTEALRREFS EL-PDGYQVQ SLPLAARRRR DNPRMVHWTG VDTVLG---- 195
KaPOx_xalh 141  EFDFSIRQHL VTEALRREFS EL-PDGYQVQ SLPLAARRRR DNPRMVHWTG VDTVLG---- 195
ScPOx_al   188  PIGPTKVGEL VLDVLRRRYG GTGPADRAPQ PMPMAVTPTP SGP--MPRTA PGTIFP---- 241
ScPOx_alh  188  PIGPTKVGEL VLDVLRRRYG GTGPADRAPQ PMPMAVTPTP SGP--MPRTA PGTIFP---- 241
PsPOx      164  AFDGAPFSDL VRERLAAVVD QGRTPAFRVQ PMPLAVHRRQ DGA--LVWSG SDVVMG---- 217
MtCarA     167  AFDASPYAGI VRERLAAVED AHRDADERVQ RMPLAVHRRD DGP--LVWSG SDVVLG---- 220
TmPOx      212  QFKESIRHNL VLNKLTEEYK GQ----RDFQ QIPLAATRR- -SPTFVEWSS ANTVFDLQNR 265

                      310        320        330        340        350        360
                ....|....| ....|....| ....|....| ....|....| ....|....| ....|....|
KaPOx      223  DLAD-GHPLF SLLPQHLCTR LVLDRDGTRI AYAEVRDLNR SETVRVVADN YVVAAGAVLA 281
ScPOx      216  PLAQGGDPAF TLLTGTLVTA LV--RDAGRV TGARLRRVAD GTESELSADT VVVCADALRT 273
KaPOx_xal  196  DLAD-GHPLF SLLPQHLCTR LVLDRDGTRI AYAEVRDLNR SETVRVVADN YVVAAGAVLA 254
KaPOx_xalh 196  DLAD-GHPLF SLLPQHLCTR LVLDRDGTRI AYAEVRDLNR SETVRVVADN YVVAAGAVLA 254
ScPOx_al   242  PLAQGGDPAF TLLTGTLVTA LV--RDAGRV TGARLRRVAD GTESELSADT VVVCADALRT 299
ScPOx_alh  242  PLAQGGDPAF TLLTGTLVTA LV--RDAGRV TGARLRRVAD GTESELSADT VVVCADALRT 299
PsPOx      218  EATR-DNPQF ELFDESLVTR VL--VEDGTA AGVEVQDRRS GDTYQVAARY VVVGADALRT 274
MtCarA     221  DITR-GNPNF TLFDESLVTR VL--VEDGRA AGVVVTDVRT GERRDVRARF VVVAADALRT 277
TmPOx      266  PNTDAPEERF NLFPAVACER VVRNALNSEI ESLHIHDLIS GDRFEIKADV YVLTAGAVHN 325
```

```
                                                                    Head domain

                                                                  Barrel shaped bottom
                                370        380        390        400        410        420
                         ....|....| ....|....| ....|....| ....|....| ....|....| ....|....|
KaPOx        282 PQLLHASGI- ---RP----- ----AALGRY LTEHPMAFCQ VILLKDLVEQ ARTDQRFGG- 327
ScPOx        274 PQLLYASGI- ---RP----- ----EALGRH LNEHAFVTAR VLLDLD---- -----RFG-- 309
KaPOx_xal    255 PQLLHASGI- ---RP----- ----AALGRY LTEHPMAFCQ VILLKDLVEQ ARTDQRFGG- 300
KaPOx_xalh   255 PQLLHASGI- ---RP----- ----AALGRY LTEHPMAFCQ VILDLD---- -----RFG-- 290
ScPOx_al     300 PQLLYASGI- ---RP----- ----EALGRH LNEHAFVTAR VLLDLD---- -----RFG-- 335
ScPOx_alh    300 PQLLYASGI- ---RP----- ----EALGRH LNEHAFVTAR VLLLKDLVEQ ARTDQRFGG- 345
PsPOx        275 PQLLWASGI- ---RP----- ----DALGRY LNDQAQVVFA SRL-RDVQP- EDAPAAANGA 319
MtCarA       278 PQLLWASGI- ---RP----- ----DALGRY LNDQAQIVFA VRM-RDFTPV VDADGVPQTG 323
TmPOx        326 TQLLVNSGFG QLGRPNPANP PELLPSLGSY ITEQSLVFCQ TVMSTELIDS VKSDMTIRGT 385

                                430        440        450        460        470        480
                         ....|....| ....|....| ....|....| ....|....| ....|....| ....|....|
KaPOx        327 ---------- ---------- -----QVARH TTLFPDD-DL PIPVDDPEPN VWIPVS-EGR 360
ScPOx        309 ---------- ---------- ---------- --LDPDALPL PRPGEFSTDS LWLPCNGPSQ 337
KaPOx_xal    300 ---------- ---------- -----QVARH TTLFPDD-DL PIPVDDPEPN VWIPVS-EGR 333
KaPOx_xalh   290 ---------- ---------- ---------- --LDPDALPL PRPVDDPEPN VWIPVS-EGR 317
ScPOx_al     335 ---------- ---------- ---------- --LDPDALPL PRPGEFSTDS LWLPCNGPSQ 363
ScPOx_alh    345 ---------- ---------- -----QVARH TTLFPDD-DL PIPGEFSTDS LWLPCNGPSQ 379
PsPOx        319 ---------- ---------- ---------- ---------- ---LSEQSGV AWVPYT-DEA 335
MtCarA       323 ---------- ---------- ---------- ---------- ---LSEYTGV TWVPFT-DDM 339
TmPOx        386 PGELTYSVTY TPGASTNKHP DWWNEKVKNH MMQHQED-PL PIPFEDPEPQ VTTLFQ-PSH 443

                                      Substrate loop
                                490        500        510        520        530        540
                         ....|....| ....|....| ....|....| ....|....| ....|....| ....|....|
KaPOx        361 PWHAQITR-- -DAFHYGDVP PHVDGRLIVD LRWFGIVEPR PDNRVTFSDT RTDVMGMPQP 417
ScPOx        338 PFHGQIMN-- -RTYVDGAGR PLAHS---VG LSLYVPVESR PQNRLVFSPG ETDLAGLPRI 391
KaPOx_xal    334 PWHAQITR-- -DAFHYGDVP PHVDGRLIVD LRWFGIVEPR PDNRVTFSDT RTDVMGMPQP 390
KaPOx_xalh   318 PWHAQITR-- -DAFHYGDVP PHVDGRLIVD LRWFGIVEPR PDNRVTFSDT RTDVMGMPQP 374
ScPOx_al     364 PFHGQIMN-- -RTYVDGAGR PLAHS---VG LSLYVPVESR PQNRLVFSPG ETDLAGLPRI 417
ScPOx_alh    380 PFHGQIMN-- -RTYVDGAGR PLAHS---VG LSLYVPVESR PQNRLVFSPG ETDLAGLPRI 433
PsPOx        336 PFHGQIMQLD ASPVPLADDD PIVPGS-IVG LGLFCAKDLQ REDRVAFDDD TRDSYGLPAM 394
MtCarA       340 PFHGQVMQLD ASPVKLADDD PAAPGS-IVG LGLFCAKDLQ ASDRVAFSDS DVDGYGMPAM 398
TmPOx        444 PWHTQIHR-- -DAFSYGAVQ QSIDSRLIVD WRFFGRTEPK EENKLWFSDK ITDAYNMPQP 500

                                                                      Catalytic His
                                550        560        570        580        590        600
                         ....|....| ....|....| ....|....| ....|....| ....|....| ....|....|
KaPOx        418 TFEYAL-SPQ DAERQHAMMA EMMRAATALG GFLPGSEPRF TAPGLPLHIA GTIRMG-DDP 475
ScPOx        392 RVEFGY-SET DRALIRRALD EVRSVAEEFG PFDPATESTV LPPGSSLHLT GTVRAGVTDD 450
KaPOx_xal    391 TFEYAL-SPQ DAERQHAMMA EMMRAATALG GFLPGSEPRF TAPGLPLHIA GTIRMG-DDP 448
KaPOx_xalh   375 TFEYAL-SPQ DAERQHAMMA EMMRAATALG GFLPGSEPRF TAPGLPLHIA GTIRMG-DDP 432
ScPOx_al     418 RVEFGY-SET DRALIRRALD EVRSVAEEFG PFDPATESTV LPPGSSLHLT GTVRAGVTDD 476
ScPOx_alh    434 RVEFGY-SET DRALIRRALD EVRSVAEEFG PFDPATESTV LPPGSSLHLT GTVRAGVTDD 492
PsPOx        395 RIHYRL-TER DHVVLDRARQ EIVRLGKAVG EPL-DERPFV LPPGASLHYQ GTTRMGETDD 452
MtCarA       399 QLHYTL-SDR DHATIDRAKA EIVRLGKAIG DPL-DDRPFV MPLGASLHYQ GTVRMGLADD 456
TmPOx        501 TFDFRFPAGR TSKEAEDMMT DMCVMSAKIG GFLPGSLPQF MEPGLVLHLG GTHRMG-FDE 559

                                                  Catalytic Asn
                                610        620        630        640        650        660
                         ....|....| ....|....| ....|....| ....|....| ....|....| ....|....|
KaPOx        476 Q--SSVVDTD SRVWGLENLY LGGNGVIPTG TACNPTLTSV AMALKAAHHL AG-------S 526
ScPOx        451 G--TGVCDPD GRVWGFDNLY LAGNGVVPTP MAANVTLTGA VTAVRTARAV TA-------R 501
KaPOx_xal    449 Q--SSVVDTD SRVWGLENLY LGGNGVIPTG TACNPTLTSV AMALKAAHHL AG-------S 499
KaPOx_xalh   433 Q--SSVVDTD SRVWGLENLY LGGNGVIPTG TACNPTLTSV AMALKAAHHL AG-------S 483
ScPOx_al     477 G--TGVCDPD GRVWGFDNLY LAGNGVVPTP MAANVTLTGA VTAVRTARAV TA-------R 527
ScPOx_alh    493 G--TGVCDPD GRVWGFDNLY LAGNGVVPTP MAANVTLTGA VTAVRTARAV TA-------R 543
PsPOx        453 G--ESVCSPD SQVWQVPGLF VAGNGVIPTA TACNPTLTSV ALAVRGARKI AEEITSSLLM 510
MtCarA       457 G--ASVCSPD SEVWGAPGLF VAGNGVIPTA TACNPTLTGV ALAVRGARHI ADEITADL-- 512
TmPOx        560 KEDNCCVNTD SRVFGFKNLF LGGCGNIPTA YGANPTLTAM SLAIKSCEYI KQNFTPSPFT 619

                                670
                         ....|....| ....|....
KaPOx        527 REARERRRTG ADEVLAVRS 545
ScPOx        502 T--------- --------- 502
KaPOx_xal    500 REARERRRTG ADEVLAVRS 518
KaPOx_xalh   484 REARERRRTG ADEVLAVRS 502
ScPOx_al     528 T--------- --------- 528
ScPOx_alh    544 T--------- --------- 544
PsPOx        511 SESDNR---- ------LSK 519
MtCarA       512 ---------- --------- 512
TmPOx        620 SEAQ------ --------- 623
```

Figure S1. Multiple sequence alignment of the bacterial dimeric *Ka*POx (UniProt A0A1E7NAU4) [12], monomeric *Sc*POx (UniProt A0A117Q443) [11], *Ps*POx (UniProt A0A024H8G7)[14] and *Mt*CarA (UniProt A0A0M2HFA3) [10] and fungal tetrameric *Tm*POx (UniProt Q7ZA32)[18]. Mutants designed in this study are included in the alignment (*Ka*POx_xal, *Ka*POx_xalh, *Sc*POx_al, *Sc*POx_alh). Important structural and catalytical features such as oligomerization motifs, substrate loop and catalytic dyad are annotated with boxes. The amino acid region T367-L384 (*Ka*POx numbering) and the glucose binding residues D369 and Y373 are highlighter with yellow and red colour, respectively in both *Ka*POx and *Ka*POx_xalh.

Table S1. Amino acid sequences of wild type enzymes *Ka*POx and *Sc*POx and variants *Ka*POx_xal, *Ka*POx_xalh, *Sc*POx_al and *Sc*POx_alh. Structurally important segments and motifs are labelled as following: oligomerization loop, arm domain, head domain, insertion-1 domain and barrel shaped bottom.

Protein	Amino acid sequence
*Ka*POx	MITRYTDTLVVGSGPVGATFARTLVESGREVLMVDAGAQLSPRPGEHLKNAYIYQHNTNLFASIIRGHLHLLSVPTSARAEL<br>AVDPAAMAELGSNRSSARNAENPDQDPYRNLSAAAACYAVGGMGTHWTGATPRHHPVLERYDGISDQEWDGLYGEAER<br>LLRVSAREFDFSIRQHLVTEALRREFSELPDGYQVQSLPLAARRRRDNPRMVHWTGVDTVLGDLADGHPLFSLLPQHLCT<br>RLVLDRDGTRIAYAEVRDLNRSETVRVVADNYVVAAGAVLAPQLLHASGIRPAALGRYLTEHPMAFCQVILLKDLVEQARTD<br>QRFGGQVARHTTLFPDDDLPIPVDDPEPNVWIPVSEGRPWHAQITRDAFHYGDVPPHVDGRLIVDLRWFGIVEPRPDNRV<br>TFSDTRTDVMGMPQPTFEYALSPQDAERQHAMMAEMMRAATALGGFLPGSEPRFTAPGLPLHIAGTIRMGDDPQSSVVD<br>TDSRVWGLENLYLGGNGVIPTGTACNPTLTSVAMALKAAHHLAGSREARERRRTGADEVLAVRS
*Sc*POx	MTHTPRTDVLIVGSGIMGSLVARLLRRSDPALHITMADGGSPIGGVPGRHLHDLDDPDLWSRYNEKVATGIQGMYTGAEVV<br>RDVAGSLPDLTPGMFHALAFGEDAEAMPQAALAWNAGGMGVHWTAATPWPAGDEVFDFGDPDAWAADLDTARRLLAVT<br>PAPIGPTKVGELVLDVLRRRYGGTGPADRAPQPMPMAVTPTPSGPMPRTAPGTIFPPLAQGGDPAFTLLTGTLVTALVRDA<br>GRVTGARLRRVADGTESELSADTVVVCADALRTPQLLYASGIRPEALGRHLNEHAFVTARVLLDLDRFGLDPDALPLPRPG<br>EFSTDSLWLPCNGPSQPFHGQIMNRTYVDGAGRPLAHSVGLSLYVPVESRPQNRLVFSPGETDLAGLPRIRVEFGYSETD<br>RALIRRALDEVRSVAEEFGPFDPATESTVLPPGSSLHLTGTVRAGVTDDGTGVCDPDGRVWGFDNLYLAGNGVVPTPMAA<br>NVTLTGAVTAVRTARAVTART
*Ka*POx_xal	MITRYTDTLVVGSGPVGATFARTLVESGREVLMVDAGAQLSPRPGEHLKNAYIYQHNTPDLWSRYNEKVATGIQGMYTGA<br>EVVRDVAGSLPDLAVGGMGTHWTGATPRHHPVLERYDGISDQEWDGLYGEAERLLRVSAREFDFSIRQHLVTEALRREFS<br>ELPDGYQVQSLPLAARRRRDNPRMVHWTGVDTVLGDLADGHPLFSLLPQHLCTRLVLDRDGTRIAYAEVRDLNRSETVRV<br>VADNYVVAAGAVLAPQLLHASGIRPAALGRYLTEHPMAFCQVILLKDLVEQARTDQRFGGQVARHTTLFPDDDLPIPVDDPE<br>PNVWIPVSEGRPWHAQITRDAFHYGDVPPHVDGRLIVDLRWFGIVEPRPDNRVTFSDTRTDVMGMPQPTFEYALSPQDAE<br>RQHAMMAEMMRAATALGGFLPGSEPRFTAPGLPLHIAGTIRMGDDPQSSVVDTDSRVWGLENLYLGGNGVIPTGTACNPT<br>LTSVAMALKAAHHLAGSREARERRRTGADEVLAVRS
*Ka*POx_xalh	MITRYTDTLVVGSGPVGATFARTLVESGREVLMVDAGAQLSPRPGEHLKNAYIYQHNTPDLWSRYNEKVATGIQGMYTGA<br>EVVRDVAGSLPDLAVGGMGTHWTGATPRHHPVLERYDGISDQEWDGLYGEAERLLRVSAREFDFSIRQHLVTEALRREFS<br>ELPDGYQVQSLPLAARRRRDNPRMVHWTGVDTVLGDLADGHPLFSLLPQHLCTRLVLDRDGTRIAYAEVRDLNRSETVRV<br>VADNYVVAAGAVLAPQLLHASGIRPAALGRYLTEHPMAFCQVILDLDRFGLDPDALPLPRPVDDPEPNVWIPVSEGRPWHA<br>QITRDAFHYGDVPPHVDGRLIVDLRWFGIVEPRPDNRVTFSDTRTDVMGMPQPTFEYALSPQDAERQHAMMAEMMRAAT<br>ALGGFLPGSEPRFTAPGLPLHIAGTIRMGDDPQSSVVDTDSRVWGLENLYLGGNGVIPTGTACNPTLTSVAMALKAAHHLA<br>GSREARERRRTGADEVLAVRS
*Sc*POx_al	MTHTPRTDVLIVGSGIMGSLVARLLRRSDPALHITMADGGSPIGGVPGRHLHDLDDNLFASIIRHLHLLSVPTSARAELAVDP<br>AAMAELGSNRSSARNAENPDQDPYRNLSAAAACYTPGMFHALAFGEDAEAMPQAALAWNAGGMGVHWTAATPWPAGD<br>EVFDFGDPDAWAADLDTARRLLAVTPAPIGPTKVGELVLDVLRRRYGGTGPADRAPQPMPMAVTPTPSGPMPRTAPGTIF<br>PPLAQGGDPAFTLLTGTLVTALVRDAGRVTGARLRRVADGTESELSADTVVVCADALRTPQLLYASGIRPEALGRHLNEHA<br>FVTARVLLDLDRFGLDPDALPLPRPGEFSTDSLWLPCNGPSQPFHGQIMNRTYVDGAGRPLAHSVGLSLYVPVESRPQNR<br>LVFSPGETDLAGLPRIRVEFGYSETDRALIRRALDEVRSVAEEFGPFDPATESTVLPPGSSLHLTGTVRAGVTDDGTGVCDP<br>DGRVWGFDNLYLAGNGVVPTPMAANVTLTGAVTAVRTARAVTART
*Sc*POx_alh	MTHTPRTDVLIVGSGIMGSLVARLLRRSDPALHITMADGGSPIGGVPGRHLHDLDDNLFASIIRHLHLLSVPTSARAELAVDP<br>AAMAELGSNRSSARNAENPDQDPYRNLSAAAACYTPGMFHALAFGEDAEAMPQAALAWNAGGMGVHWTAATPWPAGD<br>EVFDFGDPDAWAADLDTARRLLAVTPAPIGPTKVGELVLDVLRRRYGGTGPADRAPQPMPMAVTPTPSGPMPRTAPGTIF<br>PPLAQGGDPAFTLLTGTLVTALVRDAGRVTGARLRRVADGTESELSADTVVVCADALRTPQLLYASGIRPEALGRHLNEHA<br>FVTARVLLLKDLVEQARTDQRFGGQVARHTTLFPDDDLPIPGEFSTDSLWLPCNGPSQPFHGQIMNRTYVDGAGRPLAHS<br>VGLSLYVPVESRPQNRLVFSPGETDLAGLPRIRVEFGYSETDRALIRRALDEVRSVAEEFGPFDPATESTVLPPGSSLHLTG<br>TVRAGVTDDGTGVCDPDGRVWGFDNLYLAGNGVVPTPMAANVTLTGAVTAVRTARAVTART

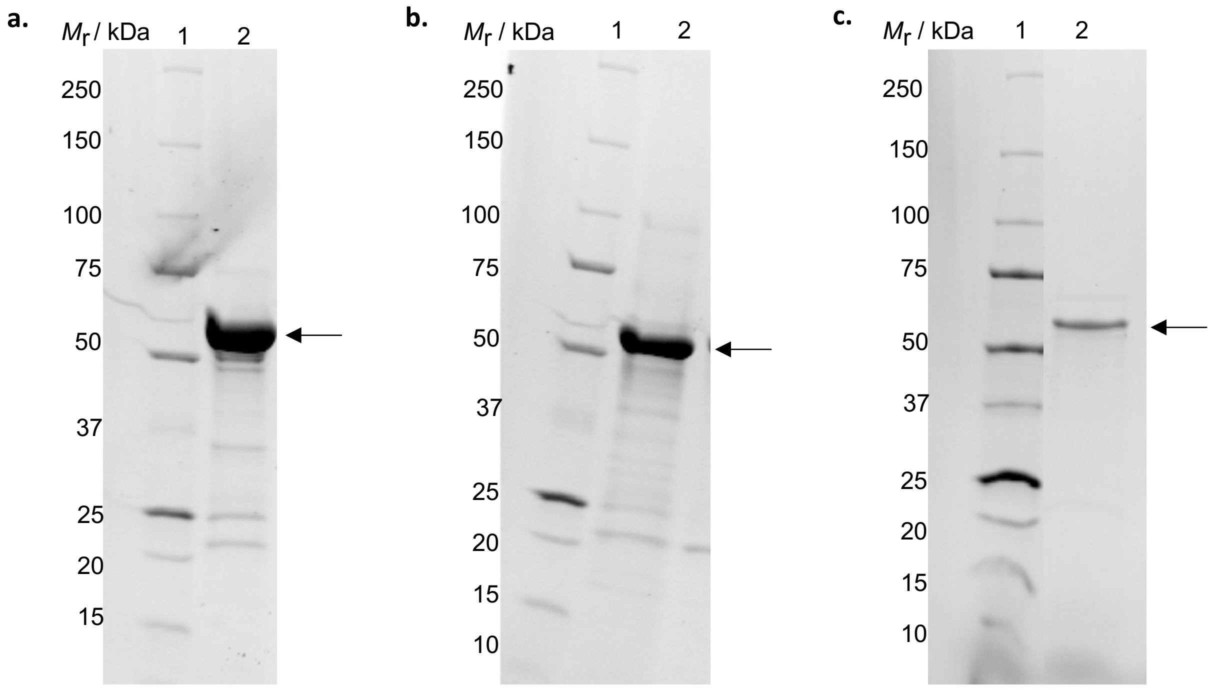

Figure S2. SDS-PAGE of pure *Ka*POx_xal and *Ka*POx_xalh in comparison with a protein ladder (BioRad, Precision Plus Protein Standard (All Blue Standards)). **a.** *Ka*POx_xal variant after purification with IMAC. 1 – protein ladder, 2 – *Ka*POx_xal. **b.** *Ka*POx_xalh variant after purification with IMAC. 1 – protein ladder, 2 – *Ka*POx_xalh. **c.** *Ka*POx_xalh variant after purification with SEC. 1 – protein ladder, 2 – *Ka*POx_xalh. Replicates not applicable. Lanes 1 and 2 in Figure S2C are spliced together.

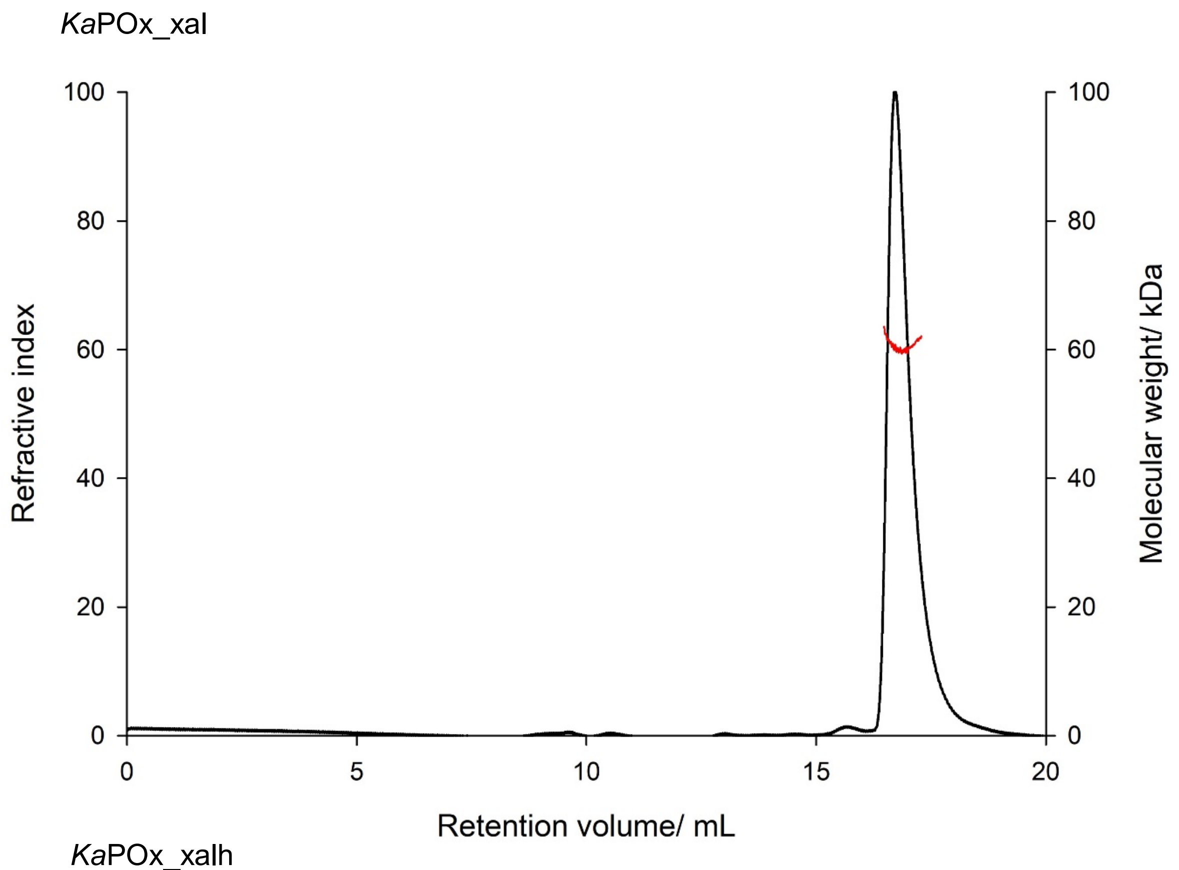

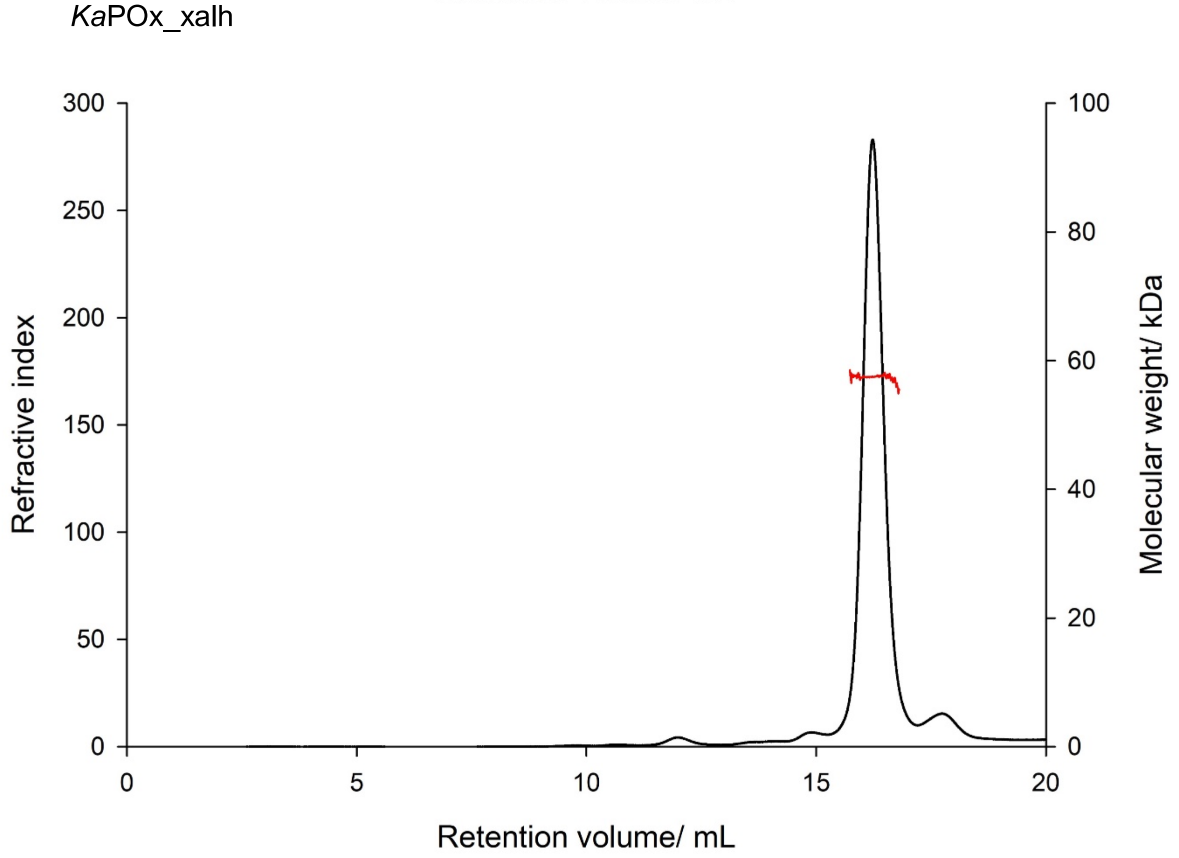

Figure S3. SEC-LS chromatograms of pure *Ka*POx_xal and *Ka*POx_xalh showing molecular weight of 61 and 57 kDa, which corresponds to monomeric state of the two mentioned variants. Replicates not applicable.

a.

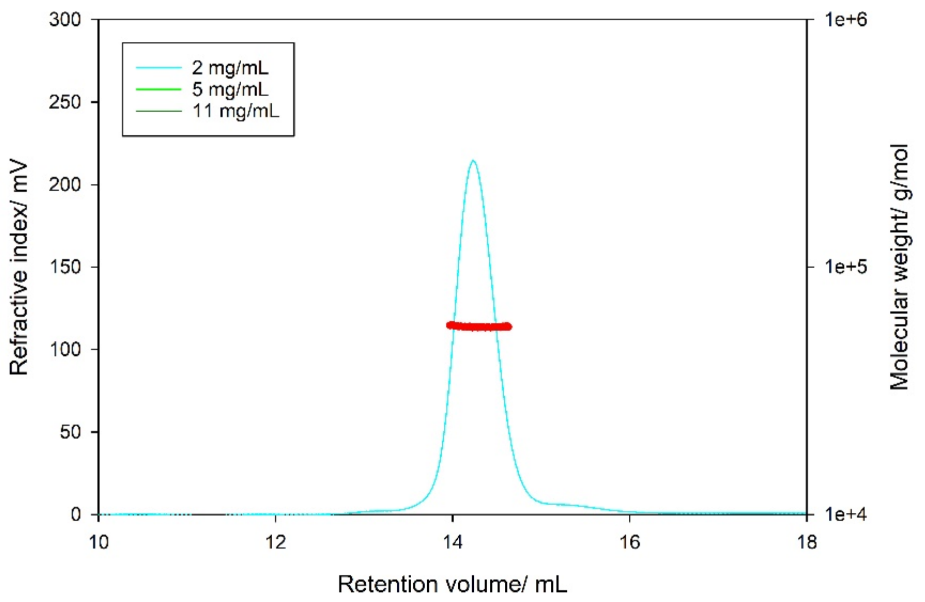

b.

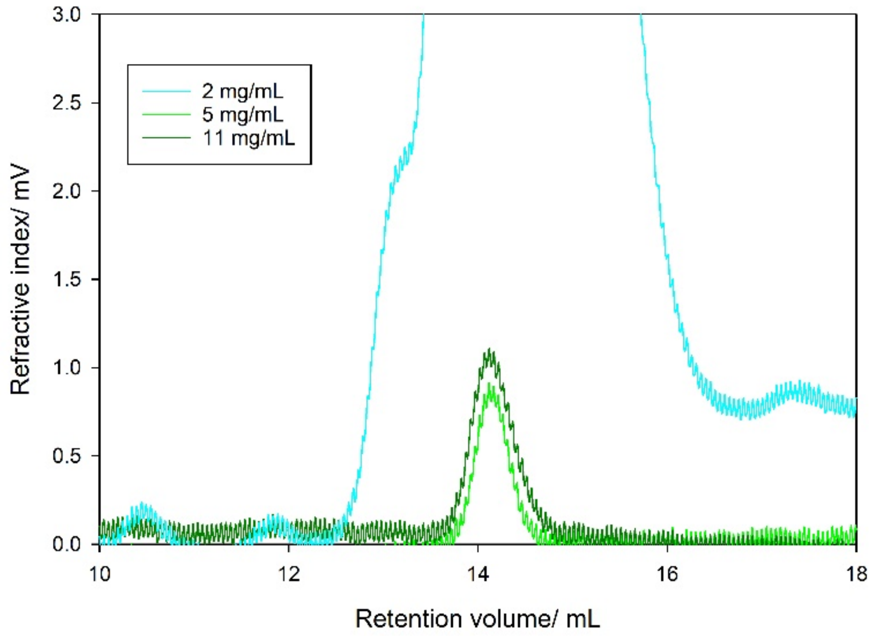

Figure S4. **a.** SEC-LS chromatograms of a *Ka*POx_xalh sample after purification in concentrations of 2, 5 and 11 mg/mL showing a molecular weight of 57 kDa, which corresponds to a monomeric state of the protein. **b.** Zooming in to the SEC-LS chromatograms of *Ka*POx_xalh of different concentrations. Replicates not applicable.

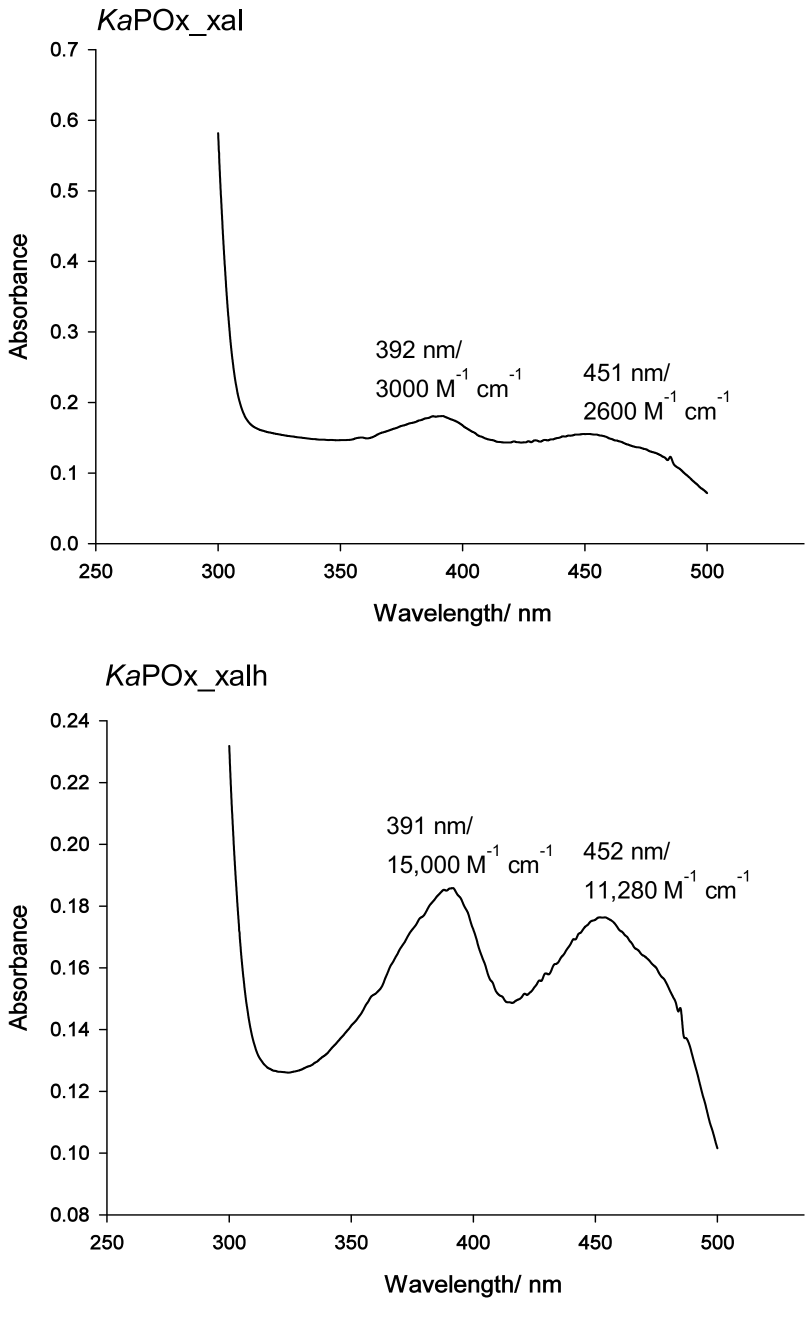

Figure S5. UV/Vis absorption spectra (300-500 nm) of *Ka*POx_xal and *Ka*POx_xalh protein samples after purification. Lambda maxima and associated extinction coefficients for the curve between 350 and 500 nm are shown. Replicates not applicable.

Table S2. Biochemical properties of the wild type *Ka*POx and its variants *Ka*POx_xal and *Ka*POx_xalh, together with information on overexpression as well as thermostability and oligomeric state (from **Figure S3**).

Protein	Number of amino acids	Molecular weight of monomer/ kDa	*E. coli* expression strain	Induction system	Yield per 1 L culture/ mg	Thermostability (T_m) / °C	Oligomeric state
*Ka*POx	556	61.2	T7 Express	Lactose, 20 h, 20°C	13.3	53*	Dimer
*Ka*POx_xal	531	58.7	T7 Express	Lactose, 20 h, 18°C	4.8	42	Monomer
*Ka*POx_xalh	515	56.9	T7 Express	Lactose, 20 h, 18°C	0.5	42	Monomer

Table S3. Structures of substrate that were oxidzed during the initial screening.

Substrate	Structure
D-glucose	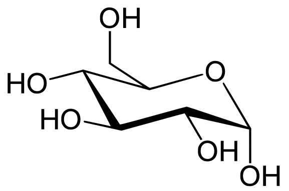
D-xylose	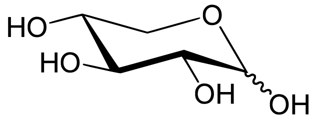
Isoorientin	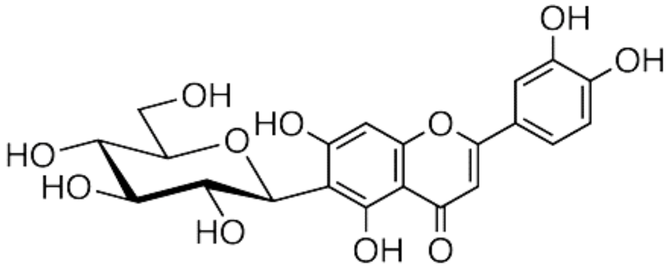
Isovitexin	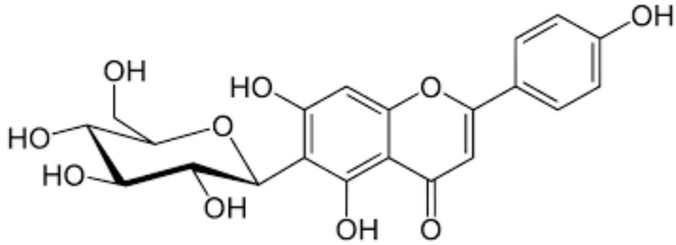
Phlorizin	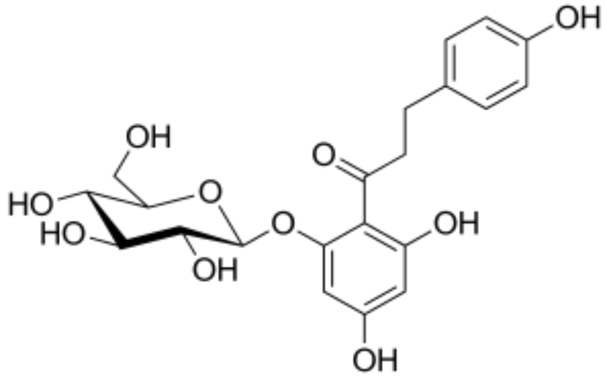

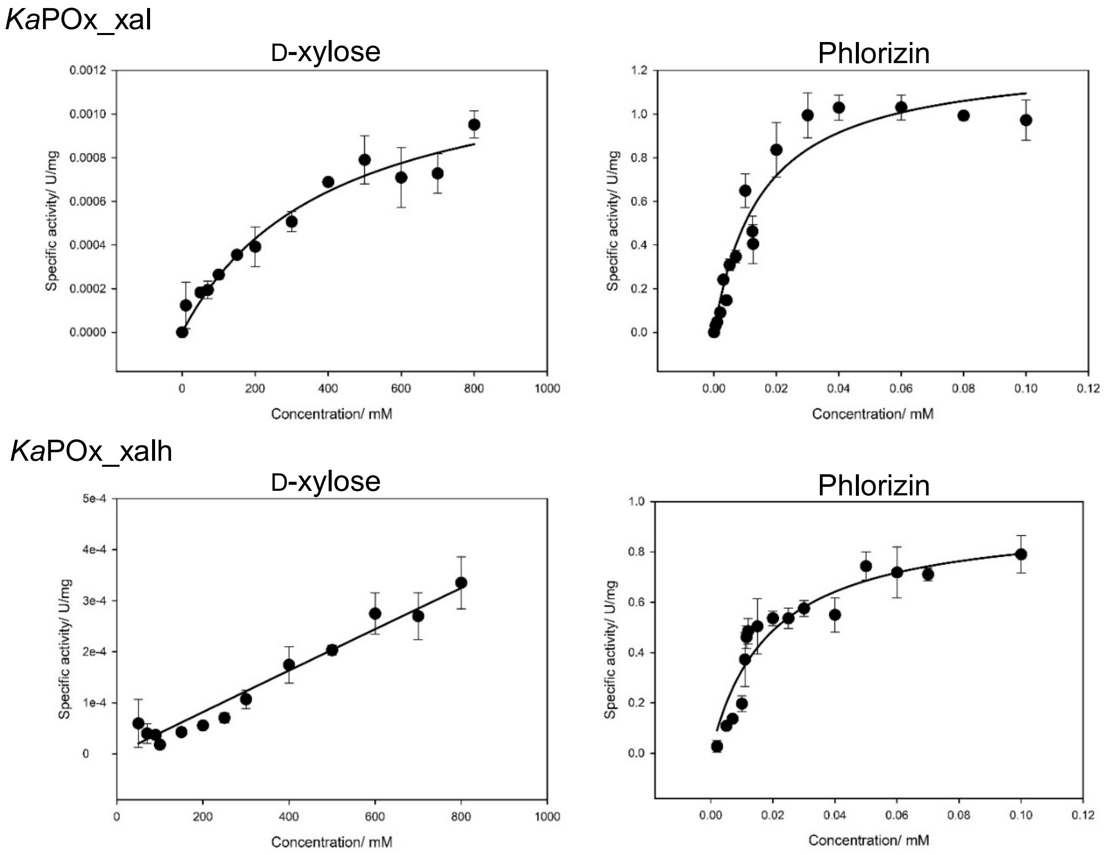

Figure S6. Michaelis-Menten curves for the variants *Ka*POx_xal and *Ka*POx_xalh. Raw data are represented with a black dot, accompanied by the fitting of the data to the Michaelis-Menten function represented by a black line. The measurements were performed in triplicates and at 30°C in 50 mM Tris-HCl, pH=7.5. Data points shown are the mean of three independent measurements ± standard deviation.

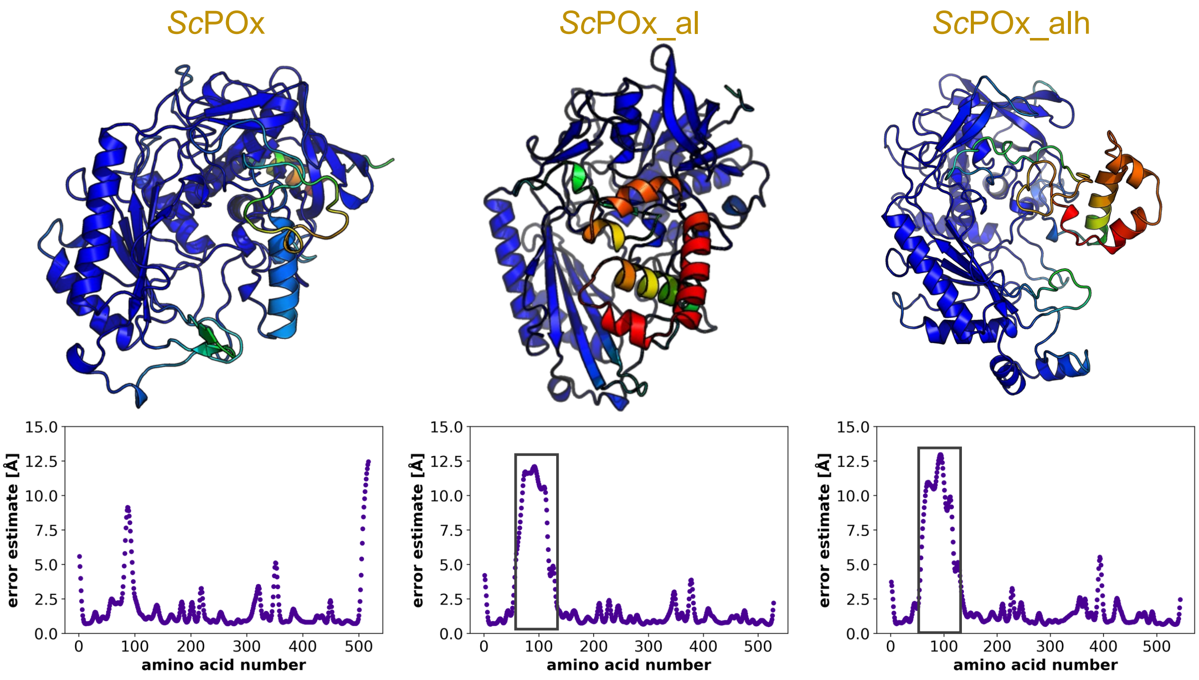

Figure S7. Error estimates in Å of each amino acid in RoseTTAFold [24] models of *Sc*POx and its variants. The structures are colored as a spectrum from blue to red, blue being lower error estimates. The added N57-Y117 oligomerization loop and arm domain (*Sc*POx_al and *Sc*POx_alh numbering) (black box) show high error estimates, and also not in a conformation to form dimers.

Table S4. Putative glucose binding residues in *Ka*POx and the *Ka*POx_xalh variant based on structural alignment of the wild-type *Ka*POx AlphaFold structure to the fungal *Pc*POx containing 3-deoxy-3-fluoro-D-glucose (PDB 4MIG) [25] and *Sc*POx. Residues in italics have a different orientation in structural models compared to *Pc*POx and wild-type *Ka*POx.

**Residue in *Pc*POx**	**Residue in *Ka*POx**	**Residue in *Ka*POx_xalh**	**Residue in *Sc*POx**
Q454	Q365	Q322	Q342
D458	D369	*D326*	R346
Y462	Y373	*Y330*	*D350*
A551	P462	P419	S436
H553	H464	H421	H438
N596	N507	N464	N482

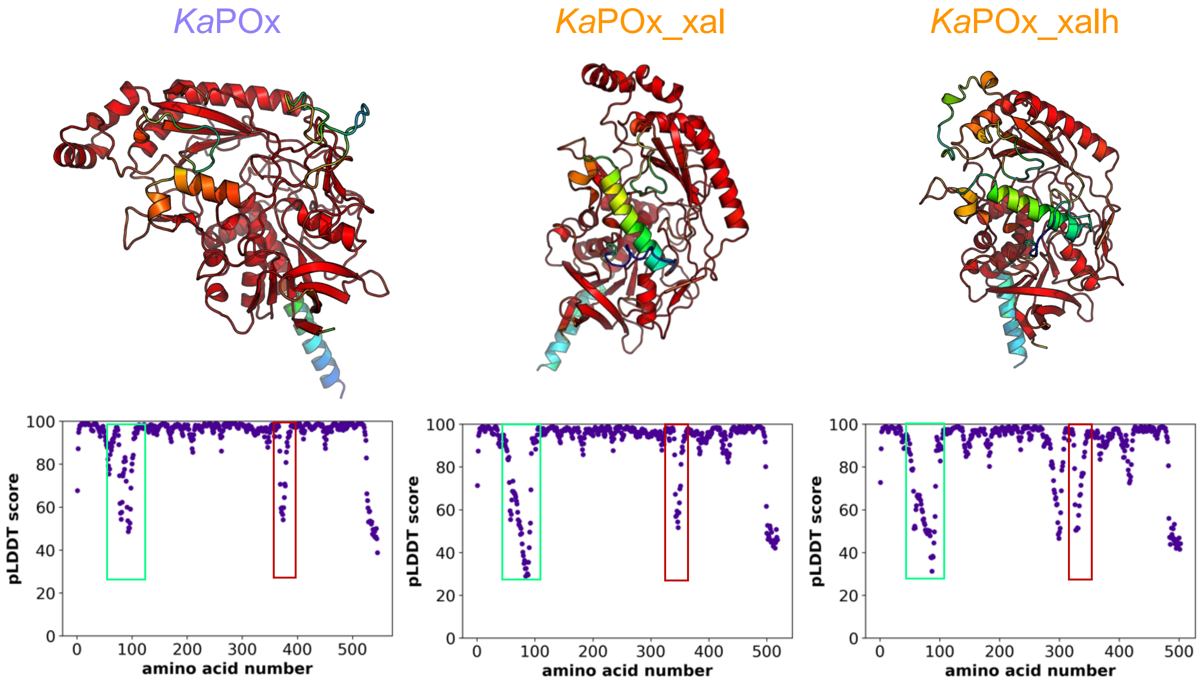

Figure S8. AlphaFold [29] models of *Ka*POx and its variants, colored in rainbow colors from red to blue (high to low) according to the pLDDT (reliability score) assigned to each amino acid, and pLDDT scores plotted against the amino acid numbers for each variant. The substrate recognition loop is marked by a red rectangle, the oligomerization loop/insertion-1 domain with a green rectangle in all plots.

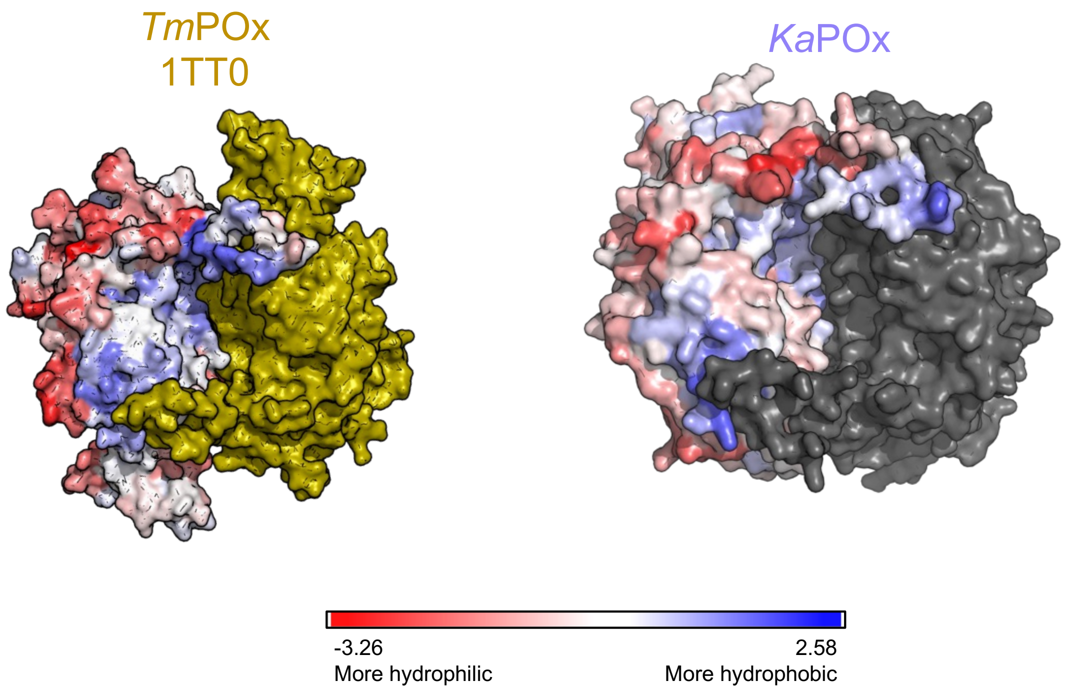

Figure S9. Comparison of the hydrophobic tetramerization surfaces of *Tm*POx (PDB 1TT0) [20] and a wild-type *Ka*POx model [12]. Tetramerization occurs through the hydrophobic surface near the hydrophobic patch and on the hydrophobic side of the arm domain in *Tm*POx. Both these regions are more hydrophilic in *Ka*POx in comparison.

Table S5. List of primers used in this study to re-clone *Sc*POx_al and *Sc*POx_alh.

Primer	Purpose	Sequence (5´- 3´)
pD441_F	Forward primer to linearize pD441 plasmid between the *Sma*I and *Hind*III restriction site	TGACAGCTCGTACCAAGCTTTAATTGGTTGTAACACTGACCCCTATTTGTTTATTTTTCTAAATACATTCAAATATGTATCC
pD441_R	Reverse primer to linearize pD441 plasmid between the *Sma*I and *Hind*III restriction site	CCCGGGCCCCTGGAACAG
pNIC_F	Forward primer to linearize pNIC-CTHO plasmid between the *Afl*II restriction site and TEV site	GCAGAGAACCTCTACTTC
pNIC_R	Reverse primer to linearize pNIC-CTHO plasmid between the *Afl*II restriction site and TEV site	AGTATATCTCCTTCTTAAGGTTAAAC
pD441_alh_F	Forward primer to amplify the *scpox_alh* gene from pET-21a and re-clone to pD441	TTCTGTTCCAGGGGCCCGGGATGACCCATACCCCGCGC
pD441_alh_R	Reverse primer to amplify the *scpox_alh* gene from pET-21a and re-clone to pD441	AAGCTTGGTACGAGCTGTCAC
pNIC_alh_F	Forward primer to amplify the *scpox_alh* gene from pET-21a and re-clone to pNIC-CTHO	CCTTAAGAAGGAGATATACTATGACCCATACCCCGCGC
pNIC_alh_R	Reverse primer to amplify the *scpox_alh* gene from pET-21a and re-clone to pNIC-CTHO	TGGAAGTAGAGGTTCTCTGCGGTACGAGCTGTCACTGCAC
pNIC_al_F	Forward primer to amplify the *scpox_al* gene from pET-21a and re-clone to pNIC-CTHO	CCTTAAGAAGGAGATATACTATGACCCATACCCCGCGTAC
pNIC_al_R	Reverse primer to amplify the *scpox_al* gene from pET-21a and re-clone to pNIC-CTHO	TGGAAGTAGAGGTTCTCTGCGGTACGGGCGGTCACTGC
